# Extracellular Nanomatrix‐Induced Self‐Organization of Neural Stem Cells into Miniature Substantia Nigra‐Like Structures with Therapeutic Effects on Parkinsonian Rats

**DOI:** 10.1002/advs.201901822

**Published:** 2019-09-30

**Authors:** Shiqing Zhang, Peng Sun, Kaili Lin, Florence Hiu Ling Chan, Qi Gao, Wai Fung Lau, Vellaisamy A. L. Roy, Hongqi Zhang, King Wai Chiu Lai, Zhifeng Huang, Ken Kin Lam Yung

**Affiliations:** ^1^ Department of Biology Hong Kong Baptist University (HKBU) Kowloon Tong Kowloon Hong Kong SAR China; ^2^ Golden Meditech Center for NeuroRegeneration Sciences HKBU Kowloon Tong Kowloon Hong Kong SAR China; ^3^ HKBU Institute of Research and Continuing Education, 9F The Industrialization Complex of Shenzhen Virtual University Park No. 2 Yuexing 3rd Road, South Zone, Hi‐tech Industrial Park, Nanshan District Shenzhen 518057 Guangdong Province China; ^4^ Department of Physics HKBU Kowloon Tong Kowloon Hong Kong SAR China; ^5^ Department of Materials Science and Engineering Southern University of Science and Technology Shenzhen 518000 Guangdong Province China; ^6^ Department of Biomedical Engineering City University of Hong Kong (CityU) Tat Chee Avenue, Kowloon Tong Kowloon Hong Kong SAR China; ^7^ Department of Materials Science and Engineering City University of Hong Kong Tat Chee Avenue, Kowloon Tong Kowloon Hong Kong SAR China; ^8^ School of Chinese Medicine HKBU Kowloon Tong Kowloon Hong Kong SAR China; ^9^ Institute of Advanced Materials State Key Laboratory of Environmental and Biological Analysis HKBU Kowloon Tong Kowloon Hong Kong SAR China

**Keywords:** extracellular nanomatrix, neural stem cells, Parkinson's disease, specific neuronal subtype differentiation, substantia nigra‐like structures

## Abstract

Substantia nigra (SN) is a complex and critical region of the brain wherein Parkinson's disease (PD) arises from the degeneration of dopaminergic neurons. Miniature SN‐like structures (mini‐SNLSs) constructed from novel combination of nanomaterials and cell technologies exhibit promise as potentially curative cell therapies for PD. In this work, a rapid self‐organization of mini‐SNLS, with an organizational structure and neuronal identities similar to those of the SN in vivo, is achieved by differentiating neural stem cells in vitro on biocompatible silica nanozigzags (NZs) sculptured by glancing angle deposition, without traditional chemical growth factors. The differentiated neurons exhibit electrophysiological activity in vitro. Diverse physical cues and signaling pathways that are determined by the nanomatrices and lead to the self‐organization of the mini‐SNLSs are clarified and elucidated. In vivo, transplantation of the neurons from a mini‐SNLS results in an early and progressive amelioration of PD in rats. The sculptured medical device reported here enables the rapid and specific self‐organization of region‐specific and functional brain‐like structures without an undesirable prognosis. This development provides promising and significant insights into the screening of potentially curative drugs and cell therapies for PD.

## Graphical abstract

Functional miniature substantia nigra‐like structures (mini‐SNLSs) with neuronal identities similar to the SN formed rapidly from neural stem cells differentiate in vitro on biocompatible silica structures sculptured into nanozigzags by glancing angle deposition. The mini‐SNLSs delivere early and progressive lesion amelioration in Parkinsonian rats. These data suggest a promising PD treatment strategy without an undesirable prognosis.

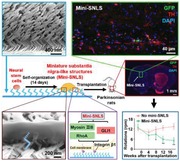

## 1 Introduction

Neurodegenerative diseases have become a primary public health threat and healthcare burden, as a consequence of the exponential growth in aging populations worldwide. Parkinson's disease (PD), a serious disorder that affects movement, is the second most common neurodegenerative disease worldwide, affecting ≈1.7% of the global population aged ≥65 years.^1^ Currently available pharmacotherapies can alleviate PD symptoms but are not curative. Additionally, chemical drugs for neurodegenerative diseases usually have severe adverse effects.^2^

The substantia nigra (SN) is a key region in the basal ganglia of the brain. PD arises from the degeneration of dopaminergic (DA) neurons in this region.^3^ Stem cell therapies and cell transplantation are considered promising strategies for the replacement of damaged cells in the SN and, ultimately, the treatment of PD. Recently, a report described the superior biofunctionality of transplanted brain organ–like structures versus transplanted neural progenitor cells.^4^ Accordingly, the construction of miniature SN‐like structures (mini‐SNLSs), which comprise mainly dopaminergic and GABAergic neurons and a small population of glutamatergic neurons, has become highly significant, given the potential uses of these structures in the screening of neurological drugs and development of curative stem cell therapies for PD.^5^ However, the actual construction of these mini‐SNLSs remains largely unexplored.

Miniature organ‐like structures that are differentiated from stem cells and mimic in vivo tissues are increasingly of interest, as they may facilitate an improved understanding of human biology and diseases.^6^, ^7^ To date, most brain‐ or midbrain‐like organoids and similar structures have been differentiated from induced pluripotent stem cells (iPSCs) via chemical and genetic manipulation.^8^, ^9^ However, these iPSC‐derived brain‐like structures usually only poorly resemble their brain counterparts, and differentiation typically requires a period of more than 1 month. Accordingly, these approaches are associated with a high risk of contamination and low efficiency of differentiation. Moreover, tumorigenicity has been reported as a clinical hurdle of iPSC‐based therapies.^10^ Alternatively, neural stem cells (NSCs) may be differentiated into the desired brain‐like structures in a direct, safe, and efficient manner.^11^ Current protocols for the formation of organ‐like structures also require the use of specific traditional chemical growth factors (GFs), such as sonic hedgehog (SHH), which is used to induce iPSC differentiation into dopaminergic and GABAergic neurons.^12^ These GFs are usually used at high concentrations to induce specific differentiation, leading to risks of carcinogenicity and tumorigenesis after transplantation in vivo.^13^, ^14^ Currently, an effective method to induce the rapid and specific differentiation of NSCs into mini‐SNLSs without applying traditional GFs is lacking. Such a method is urgently needed to enable the development of NSC therapies that may ultimately cure PD.

In this work, inorganic sculptured extracellular nanomatrices (iSECnMs) were generated by glancing angle deposition (GLAD).^15^ The associated protocol, which is biocompatible, effective, rapid, specifically controllable, and reproducible, could fulfill the urgent clinical demand for such structures. The iSECnMs are constructed from silica, a biocompatible,^16^ naturally abundant, and inexpensive substance, and are sculptured in a nanozigzag (NZ) shape to induce the rapid and specific differentiation of primary NSCs into a mini‐SNLS without requiring additional GFs. GLAD facilitates a flexible engineering process, allowing control of the material, topography,^17^ and stiffness of an iSECnM. This flexibility enables the analysis of diverse physical cues and signaling pathways that promote a fundamental understanding of iSECnM mediation and optimized differentiation. In vivo, these self‐organized mini‐SNLSs exhibited excellent survival and functionality in an animal model of PD. Ultimately, these novel biocompatible iSECnMs could be applied clinically to the specific self‐organization of functional mini‐SNLSs. These mini‐structures exhibit promise for the screening of neurological drugs and development of ultimately curative NSC therapies for PD.

## 2 Results

### 2.1 Fabrication of Silica iSECnMs

**Figure 1:**
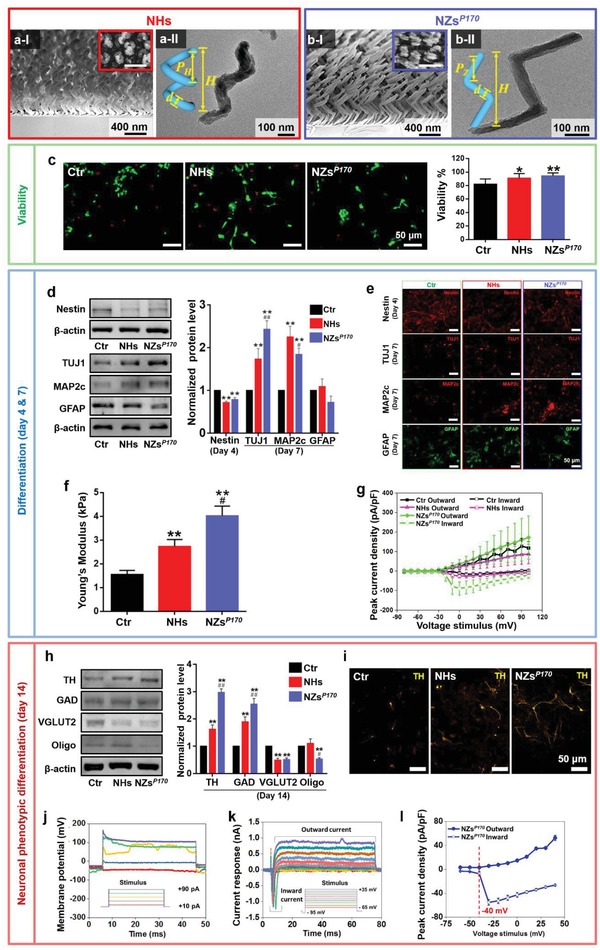
Specific phenotypic differentiation of neural stem cells (NSCs) mediated by silica inorganic sculptured extracellular nanomatrices (iSECnMs) to form miniature substantia nigra‐like structures (mini‐SNLSs). The iSECnMs were sculptured in nanohelices (NHs, with a helical pitch (*P*_H_) of ≈245 nm) and nanozigzags (NZs, with a zigzag pitch (*P*_Z_) of ≈170 nm; NZs*^P^* ^170^). a,b) Glancing angle deposition (GLAD) of the silica iSECnMs sculptured into a) NHs and b) NZs*^P^* ^170^: I) scanning electron microscopy (SEM) cross‐sectional images (insets: SEM top‐down images), scale bar: 400 nm; II) transmission electron microscopy images of individual nanostructures (insets: schemes of an NH and NZ with definitions of diverse structural parameters; scale bars: 100 nm). c) NSC viability was monitored in culture on day 7 using a live/dead assay. Fluorescent staining was performed to characterize the viability of NSCs on the iSECnMs (scale bars: 50 µm). Living and dead cells were labeled with green and red, respectively. Bar charts indicate the percentages of live cells (i.e., cell viability). d–g) Differentiation of NSCs in culture at day 4 (Nestin) and day 7 (TUJ1, MAP2c, and GFAP). d) Western blot analysis and statistical evaluation of the expression of various marker proteins. e) Immunocytochemical analysis of the differentiated NSCs on different substrates, with representative images of Nestin (stained in red), TUJ1 (stained in red), MAP2c (stained in red), and GFAP (stained in green). Scale bars: 50 µm. f) Mechanical and g) electrophysiological characterization of the differentiated cells. f) Young's modulus of the differentiated cells. g) Inward (hollow symbols) and outward (solid symbols) peak current densities of cells differentiated on different substrates: Ctr. (control glass, black squares), NHs (pink triangles), and NZs*^P^* ^170^ (green spheres). h) The specific differentiation of NSCs mediated on different substrates. A western blot analysis was used to evaluate the expression of various protein markers of differentiation on day 14: TH, GAD, VGLUT2, and Oligo. i) Immunocytochemical analysis of dopaminergic (DA) neurons induced on different substrates, with representative images of TH (stained in yellow). Scale bars: 50 µm. j–l) Electrophysiological characterization of DA neurons induced by NZs*^P^* ^170^ iSECnMs on day 14 of culture. j) Representative traces of the responses of membrane potential to step depolarization by the injection of currents ranging from 10 to 90 pA at increments of 20 pA. k) Representative traces of voltage‐dependent sodium currents in the induced DA neurons. l) Plots of the peak current density versus the voltages applied to the induced DA neurons. Upper: outward (K^+^) current density; Lower: inward (Na^+^) current density. The relative optical densities of diverse protein markers in panels (d) and (h) were assessed using β‐actin as a reference. Except for panels (f), (g), and (l), which are presented as means ± s.e.m., the data are shown as means ± s.d.; *p < 0.05 and **p < 0.01, compared with the control group; ^#^ *p* < 0.05 and ^##^ *p* < 0.01, compared with the NHs.

To date, progress in the in vitro differentiation of NSCs has mainly been attributed to the mediating effects of the extracellular matrices (ECMs) that comprise organic scaffolds,^18^ 3D stiff graphene foams,^19^ microarrays with nanotopographies,^20^ disordered nanopatterns,^21^ and carbon nanotubes.^22^ Stem cell fate decisions in vitro can be ascribed to traditional GF‐related biochemical cues and physical cues,^23^ including the material characteristics,^24^ stiffness,^25^ and topographical characteristics (e.g., crystalline structures,^26^ geometric features of nanostructures,^27^ fibrillar focal contact depth,^28^ pattern disorder,^21^ and pattern spacing^29^). In the absence of traditional GFs, physical cues intrinsic to silica iSECnMs, such as topographical characteristics and stiffness, may play an essential role in the mediatory abilities of these structures. These physical cues depend primarily on the structure of the matrix, which can be engineered flexibly using GLAD. In this study, silica iSECnMs were sculptured as vertically protruding left‐handed helices (i.e., nanohelices or NHs, **Figure** **1**a) and zigzags (i.e., NZs; Figure 1b) on a supporting substrate. The structural parameters of the iSECnMs (insets in Figure 1a‐II,b‐II), including height (*H*), wire diameter (*d*), helical pitch (*P*_H_), zigzag pitch (*P*_Z_), and pitch number (*n*), are summarized in Table S1 (Supporting Information).

The Si^4+^ cations were detected mainly X‐ray photoelectron spectroscopy (XPS; Figure S1a,b, Supporting Information), and these data revealed that the surfaces of the silica iSECnMs were oxidized stoichiometrically and that the as‐deposited nanostructures had amorphous structures (Figure S1c, Supporting Information). Typically, the NZs exhibited a groove‐like topography (insets in Figure 1b‐I) due to GLAD‐induced anisotropic NZ growth,^17^ and were markedly different from NHs which exhibited a helical topography (inset in Figure 1a‐I). The groove‐like topography of the former structures became evident as the *P*_Z_ elongated. The stiffnesses of the silica iSECnMs were measured statistically using atomic force microscopy (AFM; Figure S1d, Supporting Information) in terms of Young's modulus, and are summarized in Table S1 (Supporting Information). Two‐pitch NHs (*P*_H_ ≈ 245 nm) had a Young's modulus comparable to that of three‐pitch NZs (*P*_Z_ ≈ 170 nm, i.e., NZs*^P^* ^170^), and the stiffness of three‐pitch NZs increased as the *P*_Z_ increased from 80 to 225 nm. These two‐pitch NHs and three‐pitch NZs*^P^* ^170^, which had a similar stiffness but distinct topographies, were selected for a study of the in vitro mediation of NSC differentiation.

### 2.2 Silica iSECnM‐Mediated Self‐Organization of the Mini‐SNLS

Isolated NSCs were mediated in vitro on silica iSECnMs in commercial neurobasal medium containing fetal bovine serum (FBS) to maintain cell health in culture, and the viability and differentiation of the cells were evaluated.^30^ No additional GFs were added to the cultures. Similar levels of NSC viability (monitored using a live/dead assay; Figure 1c) were observed with NHs and NZs*^P^* ^170^, and both structures were evidently superior to the conditions on glass plates (i.e., the control group). This finding illustrates that iSECnMs can promote NSC viability.

Regarding the in vitro differentiation of NSCs, iSECnMs induced significant decreases in the expression of Nestin protein (an NSC marker) on day 4 (nearly independent on the sculptured shape), compared with the control group. Moreover, growth on iSECnMs led to significant increases in TUJ1 (a marker of maturing neurons)^31^ and MAP2c (involved in synaptogenesis and is downregulated in later stages of neuronal development)^32^ proteins on day 7 (Figure 1d). Higher TUJ1 protein expression was observed on NZs*^P^* ^170^ than on NHs, whereas the opposite pattern was observed for MAP2c expression. In other words, NZs*^P^* ^170^ induced NSCs differentiation more rapidly than NHs and the control group. Meanwhile, NZs*^P^* ^170^ tended to reduce the expression of glial fibrillary acidic protein (GFAP) (an astrocyte marker)^19^ relative to the levels in the control group and cells exposed to NHs (Figure 1d), illustrating that NZs*^P^* ^170^ tend to suppress the differentiation of NSCs into astrocytes. The immunofluorescence staining results (Figure 1e) are consistent with the western blot results (Figure 1d). Previous studies found that mediation by GFs and 3D nanofibrous scaffolds led to the differentiation of NSCs into both neurons and astrocytes.^33^, ^34^ Silica NZs are superior to the existing techniques used to induce the preferential differentiation of NSCs into neurons.

Cell stiffness is considered a positive index of the maturation of a differentiated cell.^35^ AFM was used to locate (Figure S2a, Supporting Information) and evaluate the stiffness (i.e., Young's modulus) of the differentiated neurons. A statistical evaluation (Figure S2b, Supporting Information) revealed that the Young's modulus of the neurons differentiated under various conditions evidently increased in the following order: control glass, NHs, and NZs*^P^* ^170^ (Figure 1f).

Patch clamp recording was used to study the electrophysiological behavior of the differentiated neurons. The voltage‐clamp data revealed that step‐voltage stimuli ranging from −80 to 100 mV significantly elicited both the inward and outward ionic currents of the differentiated neurons (Figure S2c, Supporting Information). The activities of the Na^+^ and K^+^ ion channels and ion pumps can be ascribed to neuronal signaling.^36^ The rapid inward current (Figure S2d, Supporting Information) indicated voltage‐activated current in the Na^+^ channels, a significant characteristic of neuronal cells.^37^ In contrast, the astrocytes only harbored K^+^‐ion channels. The peak current density (i.e., peak current amplitude per unit cell capacitance; unit cell capacitance is proportional to the cell surface area) on the silica iSECnMs was evaluated as a function of the step‐voltage stimulus. The inward peak current density in the neurons differentiated on NZs*^P^* ^170^ represents the characteristics of the rapid Na^+^ ionic current and delayed‐rectifier K^+^ ionic current,^38^ and these parameters could not be observed clearly on NHs or the control glass (Figure 1g). Furthermore, neurons differentiated on NZs*^P^* ^170^ exhibited axon‐like structures (indicators of functional neurons)^39^ that were markedly thicker than those observed on neurons differentiated on control glass and NHs (marked by white arrows in Figure S2a in the Supporting Information). The rapid maturation of neurons on NZs*^P^* ^170^ is a highly desirable characteristic for transplantation, as it would reduce the required culture time and minimize the medical and contamination risks.

When the iSECnM mediation period was extended to 14 days, the NZs*^P^* ^170^ significantly amplified the expression of tyrosine hydroxylase (TH; a dopaminergic neuron marker, consistent with the results of TH immunocytochemistry; Figure 1i) and glutamic acid decarboxylase (GAD) (a marker of GABAergic neurons, which can strengthen the functions of dopaminergic neurons and SN),^40^ relative to the levels observed in the control group and NHs (Figure 1h). Furthermore, NZs*^P^* ^170^ remarkably suppressed the expression of VGLUT2 (a glutamatergic neuron marker) and Oligo (an oligodendrocyte marker; Figure 1h). These findings emphasize that mediation with NZs*^P^* ^170^ induces the specific phenotypic differentiation of neurons to enable a mini‐SNLS self‐organization similar to that of the SN in the brain. The functionality of the self‐organized mini‐SNLS was monitored via a study of the electrophysiological characteristics of the differentiated dopaminergic neurons. Notably, the NSCs exhibited single spontaneous firing until day 14 (Figure 1j). Individual neurons differentiated on the NZs*^P^* ^170^ exhibited voltage‐gated Na^+^ and K^+^ ionic currents in the whole‐cell patch‐clamp mode (Figure 1k), with rapid inactivation of inward (Na^+^ channels) and activation of outward (K^+^ channels) currents at −40 mV in the voltage‐clamp mode (Figure 1l). These electrophysiological characteristics were consistent with those of iPSC‐induced dopaminergic neurons,^41^ and thus verified the dopaminergic functionality of the differentiated neurons. These findings clearly illustrate that NZ mediation most effectively induces the differentiation of NSCs into functional dopaminergic and GABAergic neurons while prohibiting differentiation into oligodendrocytes and glutamatergic neurons. In other words, NZ mediation best induces the self‐organization of a mini‐SNLS that is similar to the SN in an actual brain. This rapid NZ‐induced self‐organization occurred within 2 weeks, a shorter duration than that reported for iPSC mediation.^8^, ^9^

**Figure 2:**
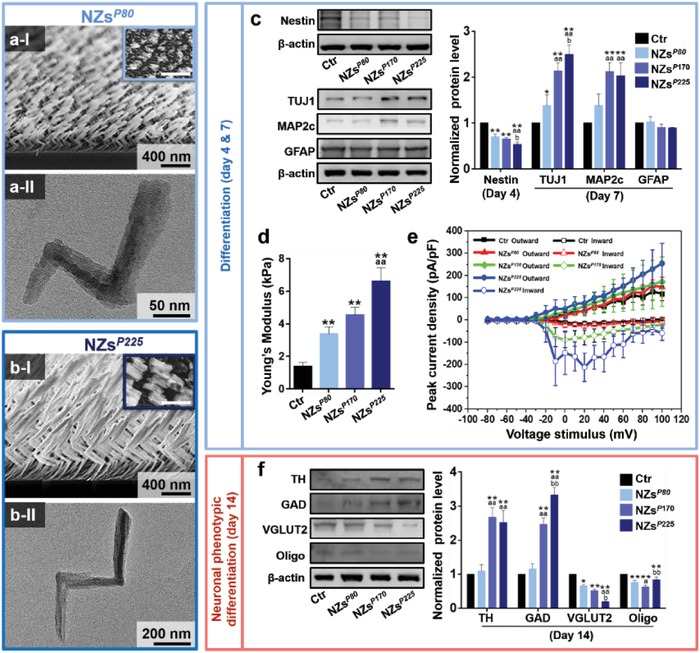
Specific phenotypic differentiation of neural stem cells (NSCs) mediated with a series of silica nanozigzags (NZs) and the formation of miniature substantia nigra‐like structures (mini‐SNLSs). The NZs were sculptured using zigzag pitches (*P*_Z_) of ≈80 (i.e., NZs*^P^* ^80^), ≈170 (i.e., NZs*^P^* ^170^), and ≈225 nm (i.e., NZs*^P^* ^225^). a,b) Glancing angle deposition (GLAD) of silica inorganic sculptured extracellular nanomatrices (iSECnMs) sculptured into the NZs with *P*_Z_ values of a) ≈80 and b) ≈225 nm: I) scanning electron microscopy (SEM) cross‐sectional images (insets: SEM top‐down images), scale bars: 400 nm; II) transmission electron microscopy images of individual NZs, scale bar: a‐II) 50 nm and b‐II) 200 nm. c) The differentiation of NSCs mediated on NZs with different *P*_Z_ values was determined using a western blot analysis of the expression of various protein markers of differentiation: Nestin (day 4), TUJ1 (day 7), MAP2c (day 7), GFAP (day 7). d) Young's modulus of the differentiated cells. e) Inward (hollow symbols) and outward (solid symbols) peak current densities of cells differentiated on different substrates: Ctr. (black squares), NZs*^P^* ^80^ (red triangles), NZs*^P^* ^170^ (green spheres), and NZs*^P^* ^225^ (blue spheres). f) The specific differentiation of NSCs mediated on NZs with different *P*_Z_ values was determined on day 14, using a western blot analysis of the expression of various protein markers of differentiation: TH, GAD, VGLUT2, and Oligo. The relative optical densities of diverse protein markers in panels (c) and (f) were assessed using β‐actin as a reference. Except for panels (d) and (e), which are presented as the means ± s.e.m., the remaining data are shown as means ± s.d.; *p < 0.05 and **p < 0.01, compared with the control group; ^a^ *p* < 0.05 and ^aa^ *p* < 0.01, compared with the NHs*^P^* ^80^; ^b^ *p* < 0.05 and ^bb^ *p* < 0.01, compared with the NHs*^P^* ^170^.

GLAD enables the flexible engineering of *P*_Z_ (a characteristic helical parameter of NZs) and thus enables control of the NZ‐mediated self‐organization of the mini‐SNLS. The elongation of *P*_Z_ from 80 (i.e., NZs*^P^* ^80^; **Figure** **2**a) to 225 nm (i.e., NZs*^P^* ^225^; Figure 2b) tended to promote the differentiation of NSCs into neurons and prohibit differentiation into astrocytes (Figure 2c). The Young's modulus (Figure 2d) and electrophysiological properties (Figure 2e) of the differentiated neurons tended to increase gradually with the elongation of *P*_Z_. After a 14 day culture, *P*_Z_ elongation increased the expression of TH to a maximum level at a *P*_Z_ of 170 nm and also gradually increased the expression of GAD, but suppressed the expression of VGLUT2 and Oligo (Figure 2f). These findings comprehensively demonstrate that the elongation of *P*_Z_ from 80 to 225 nm favors the rapid differentiation of NSCs into a functional mini‐SNLS.

Notably, poor premelting of the silica targets, an unavoidable phenomenon that occurs during electron‐beam evaporation, causes fluctuations in the deposition rate during GLAD that become increasingly severe as the deposition is elongated. A further elongation of the *P*_Z_ above 225 nm caused a serious and unrepeatable deposition of silica NZs on the supporting substrate over a macroscale area. This phenomenon currently prohibits a comprehensive study of the effect of *P*_Z_ elongation on NZ mediation. However, these findings clearly demonstrate that NSC differentiation can be engineered effectively by tailoring the sculptured nanostructures.

### 2.3 Physical Cues of Silica iSECnMs

**Figure 3:**
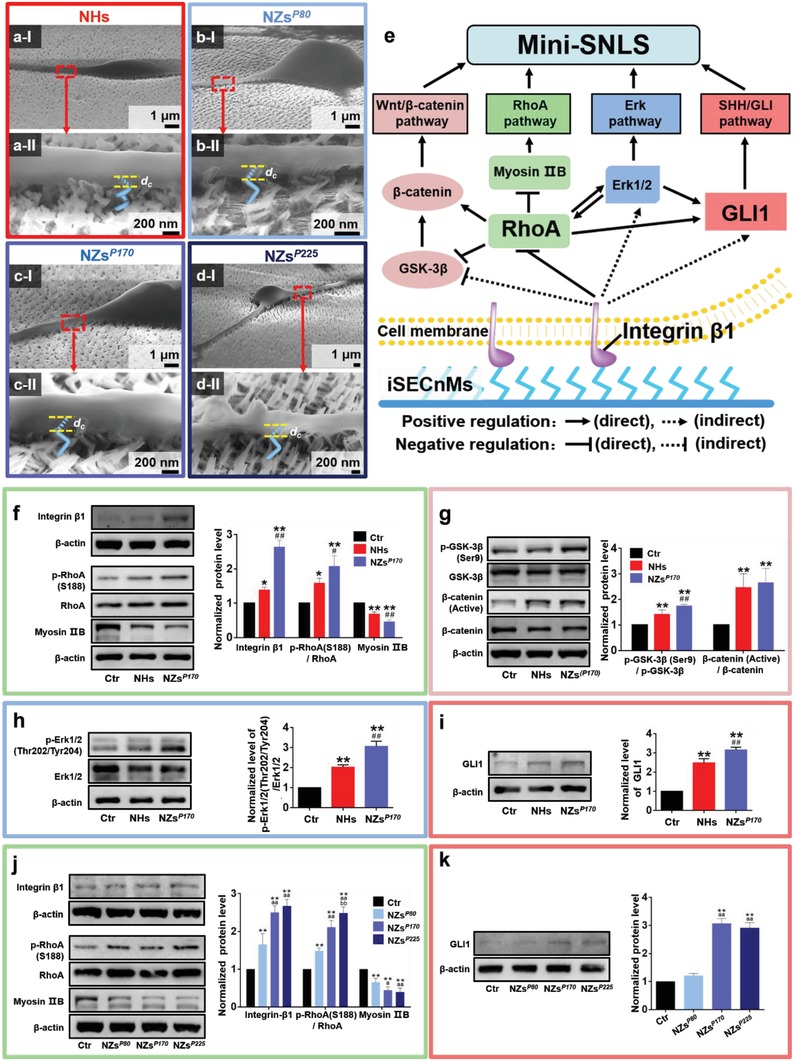
Mediation by silica inorganic sculptured extracellular nanomatrices (iSECnMs) is driven by physiological cues and multiple signaling pathways. Differentiation of neural stem cells (NSCs) on day 7 in culture via a) nanohelices (NHs), b) nanozigzags with a zigzag pitch of 80 nm) NZs*^P^* ^80^, c) NZs*^P^* ^170^, and d) NZs*^P^* ^225^. a–d) Cross‐sectional scanning electron microscopy images; II) yellow dashed lines indicate the contact depths (*d*_c_ values) at the interfaces of the differentiated cells and iSECnMs. Scale bar: I) 1 µm and II) 200 nm. e) An overview of the multiple signaling pathways stimulated in NSCs by silica iSECnMs. f–i) Analysis of multiple signaling pathways stimulated on control glass, NHs, and NZs*^P^* ^170^, as determined by western blot analyses and the relative optical densities of various markers of different signaling pathways: f) integrin β1, p‐RhoA(S188), RhoA, and Myosin IIB (integrin β1/RhoA/Myosin IIB pathway); g) β‐catenin (active), β‐catenin, p‐GSK‐3β (Ser9), and GSK‐3β (Wnt/β‐catenin pathway); h) p‐Erk1/2 (Thr202/Tyr204) and Erk1/2 (Erk pathway); and i) GLI1 (GLI1 pathway). *p < 0.05 and **p < 0.01, compared with the control group; ^#^ *p* < 0.05 and ^##^ *p* < 0.01, compared with NHs. j,k) Analysis of multiple signaling pathways activated by NHs*^P^* ^80^, NZs*^P^* ^170^, and NZs*^P^* ^225^, as determined by western blot analyses and the relative optical densities of various markers of different signaling pathways: j) integrin β1, p‐RhoA(S188), RhoA, and Myosin IIB (integrin β1/RhoA/Myosin IIB pathway) and k) GLI1 (GLI1 pathway). **p < 0.01, compared with the control group; ^a^ *p* < 0.05 and ^aa^ *p* < 0.01, compared with the NHs*^P^* ^80^; ^bb^ *p* < 0.01, compared with the NHs*^P^* ^170^. The relative optical densities of various protein markers were analyzed using β‐actin as a reference. Data are shown as means ± s.d.

In the absence of chemical manipulation, NSC differentiation can be induced by the physical stimulation of ECMs and subsequent activation of multiple signaling pathways. Scanning electron microscopy (SEM) revealed that the differentiated neuronal cells appeared to spread over and strongly adhere to the sculptured nanostructures (**Figure** **3**a–d), consistent with a previous report describing cellular interactions with 2D ECMs at the cell bases.^42^ The formation of fibrillar focal contacts enabled the spreading neuronal cells to wrap strongly around the top portions of the iSECnMs. The similar levels of stiffness of the two‐pitch NHs (Figure 3a) and three‐pitch NZs*^P^* ^170^ (Figure 3c; Figure S1d, Supporting Information) suggest that NSCs physically perceive the topography of the iSECnMs. In other words, the NZ‐promoted self‐organization of the mini‐SNLS can be attributed to topographic cues. These topographic cues, which include the geometrical profile of the cell–matrix contacts and the contact depth (*d*_c_) of the cells, were reported to affect NSC differentiation.^43^ The NHs exhibited a helical profile at the cell–matrix contacts (inset of Figure 1a‐I), whereas the NZ arrays exhibited uniquely disordered, groove‐like topographies (insets of Figures 1b‐I and 2a‐I,b‐I). More favorable NSC differentiation has been observed on nanopatterned groove‐like substrates,^44^ consistent with the observed results of NZ mediation. This outcome may be attributed to the ability of the topographic grooves to enhance focal adhesion and promote increased physical contact between the growing cells and sculptured nanostructures, especially once the neurites expand to the spaces in the grooves.^45^ Moreover, a large *d*_c_ favors the differentiation of human mesenchymal stem cells specifically to the osteoblast lineage, via the development of a high level of cellular organization.^28^ The *d*_c_ values of various silica iSECnMs were characterized by SEM and are summarized in Table S1 (Supporting Information). These values were roughly equal to a half‐pitch of the NHs (Figure 3a‐II) and single pitch of the NZs (Figure 3b–d‐II). The differentiating NSCs perceived a *d*_c_ of 192 ± 4 nm on NZs*^P^* ^170^, which was larger than the corresponding value of 146 ± 4 nm on NHs. Given this groove‐like topography, an elongation of *P*_Z_ from 80 to 225 nm increased both the *d*_c_ from 80 to 255 nm and the stiffness of the NZs from 0.62 to 7.85 GPa (Figure S1d, Supporting Information), consistent with previous reports in which a high level of ECM stiffness was favorable for cell differentiation.^46^ Comprehensively, this groove‐like topography, large *d*_c_, and high matrix stiffness account for the ability of NZs to facilitate the self‐organization of mini‐SNLSs.

### 2.4 Silica iSECnM‐Activated Signaling Pathways

Multiple signaling pathways contribute to the induction of NSC differentiation and the self‐organization of mini‐SNLSs, as shown in Figure 3e. As NSC differentiation is primarily induced by physical stimulation via the iSECnMs, integrin β1‐mediated signaling via its downstream effectors, RhoA and Myosin IIB, is responsible for regulating the cellular response to extracellular mechanical forces and is the key pathway that mediates the mechanisms by which iSECnMs induce NSC differentiation. In this study, the extracellular physical cues of silicon iSECnMs activated the integrin β1/RhoA/Myosin IIB pathway in the following order: control glass, NHs, and NZs*^P^* ^170^ (Figure 3f). Elongation of the *P*_Z_ from 80 to 225 nm significantly enhanced the activation of integrin β1/RhoA/Myosin IIB (Figure 3j), consistent with an increase in the self‐organization of mini‐SNLSs with *P*_Z_ elongation. Conversely, iSECnMs composed of titanium oxides (TiO*_x_*) sculpted into NHs and NZs (Figure S3 and Table S1, Supporting Information) could not activate the integrin β1/RhoA/Myosin IIB pathway effectively, and thus could not induce NSC differentiation (Figures S3 and S4, Supporting Information). These findings demonstrate that the integrin β1/RhoA/Myosin IIB pathway is a key factor in the iSECnM‐stimulated self‐organization of the mini‐SNLS.

Consequently, several other intercellular signaling pathways are regulated to promote the differentiation of NSCs into specific neuronal subtypes. In this study, the physical cues of NZs*^P^* ^170^ induced the activation of GSK‐3β/β‐catenin (Wnt/β‐catenin pathway; Figure 3g) and Erk1/2 (Erk pathway; Figure 3h) in a superior manner, but did not affect Akt (Akt pathway; Figure S5, Supporting Information). Moreover, these physical cues eventually induced the upregulation of GLI1 (a key transcription factor in the SHH/GLI1 pathway; Figure 3i) in the absence of traditional GFs (Section S4, Supporting Information). Therefore, the silica NZs induced GLI1 expression that mimicked the GF effect of SHH, thus stimulating the differentiation of NSCs into dopaminergic and GABAergic neurons and the eventual formation of mini‐SNLSs.^47^ Similarly, the elongation of *P*_Z_ from 80 to 225 nm significantly activated the SHH/GLI1 signaling pathway, the activity of which plateaued at *P*_Z_ values of >170 nm (Figure 3k). In other words, protein effectors could be tuned via engineerable iSECnM‐associated physical cues to stimulate the desired neuronal phenotypes. Through physical cues such as the groove profiles and *d*_c_ values of the cell–matrix contacts and the matrix stiffness, silica iSECnMs activate the integrin β1–RhoA–GLI1 signaling pathway to stimulate the specific self‐organization of mini‐SNLSs in the absence of additional GFs.

### 2.5 Therapeutic Effect of the Self‐Organized Mini‐SNLS on a Rat Model of PD

**Figure 4:**
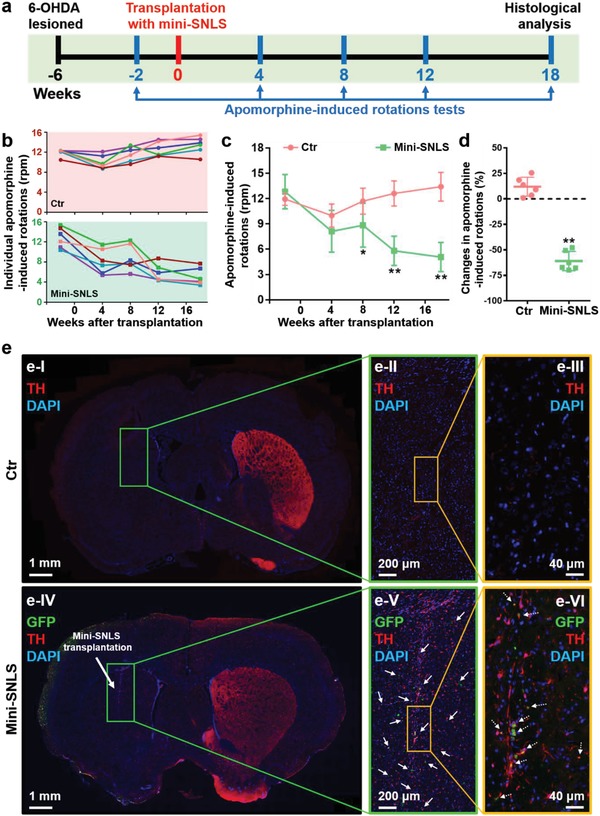
Therapeutic effects of miniature substantia nigra‐like structures (mini‐SNLSs) in a rat model of 6‐OHDA‐lesioned Parkinson's disease. a) A schematic representation of mini‐SNLS transplantation and the behavioral test procedure. b) Individual apomorphine‐induced rotations of rats without (control rats, Ctr; pink background) and with transplanted mini‐SNLSs (mini‐SNLS rats; green background) as a function of time. c) Statistical analysis of apomorphine‐induced rotations in the Ctr (pink) and mini‐SNLS (green) rats. d) Changes in the apomorphine‐induced rotations of the Ctr (pink) and mini‐SNLS (green) rats during the 18th week post‐transplantation. Data are shown as means ± s.d.; *p < 0.05 and **p < 0.01, compared with the Ctr rats. e) Immunohistochemical analysis of the brain coronal sections in I–III) Ctr rats and IV–VI) mini‐SNLS rats at 18th week post‐transplantation: TH (red), GFP (green), and DAPI (blue). The box in each image outlines the area magnified to the right. Grafted cells (solid arrows in panel (e‐V)) and double‐labeled cells (dotted arrows in panel (e‐VI)) were distributed widely around the primary transplantation site.

In a 2 week culture, NZ*^P^* ^225^ mediation induced the differentiation and maturation of NSCs into mini‐SNLSs. The latter structures exhibited good survival and phenotypic stability after transplantation into adult rat brains (Figures S6 and S7 and Section S5, Supporting Information). These results thus enabled a study of the therapeutic effects of these self‐organized mini‐SNLSs in rat models of PD. In each rat model, PD was induced by using 6‐hydroxydopamine (6‐OHDA) to generate a unilateral lesion on the left side of the medial forebrain bundle (MFB).^48^ The NSCs were differentiated on silica NZs*^P^* ^225^ for 14 days, after which the self‐organized mini‐SNLSs were labeled with green fluorescent protein (GFP) and transplanted into the striatum of each PD rat.^49^ Apomorphine‐induced contralateral rotation tests were performed to evaluate the in vivo therapeutic efficacy of mini‐SNLS transplantation in this PD model (**Figure** **4**a). In the second week before transplantation, all rats exhibited severe apomorphine‐induced motor asymmetry. Subsequently, all mini‐SNLS‐transplanted rats exhibited a progressive reduction in apomorphine‐induced rotations during the 18 week period after transplantation. In contrast, rats not transplanted with mini‐SNLSs (i.e., control [Ctr] rats) did not exhibit improvements in motor asymmetry during the testing period (Figure 4b–d). In the transplanted group, the significant reductions in apomorphine‐induced rotations emerged rapidly, beginning on the eighth week after mini‐SNLS transplantation (Figure 4c). In the 18th week post‐transplantation, the TH^+^ signal at the striatum was nearly undetectable in the lesioned (left) side versus the contralateral side (right) in the control rats (Figure 4e‐I–e‐III). In contrast, the striatal graft area in each PD rat comprised both GFP^+^ and TH^+^ cells, as well as many GFP^+^ and TH^+^ double‐labeled cells corresponding to neurite‐like structures which were widely distributed around the primary transplantation site (Figure 4e‐IV–e‐VI). Additionally, no tumor‐like and tumorigenic characteristics were detectable by hematoxylin and eosin (H&E) staining in 18th week post‐transplantation (Figure S8, Supporting Information). These results illustrate that the silica NZ‐mediated, self‐organized mini‐SNLSs induced early and positive therapeutic effects in 6‐OHDA lesion‐induced PD rats.

## 3 Discussion

Our findings provide a foundation upon which the silica iSECnM‐based procedure can be optimized to create NSC‐derived mini‐SNLSs with an excellent graft survival capacity in vivo and good behavioral outcomes in a murine model of PD. Silica iSECnMs sculptured into the NZs by GLAD appear to be superior for specific neuronal phenotypic differentiation and the rapid self‐organization of functional mini‐SNLSs, which is promoted by activation of the integrin β1–RhoA–GLI1 signaling pathway. This process is induced by the physical cues of the NZs. Unlike NHs, silica NZs exhibit a unique groove‐like topography that strongly enhances cell viability and promotes specific neuronal phenotypic differentiation. Elongation of the *P*_Z_ from 80 to 225 nm increases the stiffness of the NZs and provides a longer contact depth, thus effectively promoting the self‐organization of the mini‐SNLS. As *P*_Z_ elongation facilitates the differentiation of cells with neuronal phenotypes from NSCs, the use of a *P*_Z_ of >225 nm for rapid NSC differentiation is urgently needed. This concept is currently being studied in experiments involving stabilization of the silica deposition rate during GLAD to enable the repeatable macroscale generation of silica NZs on a supporting substrate.

This study has demonstrated for the first time that biocompatible iSECnMs enable the rapid self‐organization of functional miniature brain‐like structures (i.e., mini‐SNLSs) derived from naïve primary NSCs. Moreover, these mini‐SNLSs definitively delivered early and progressive amelioration of the motor symptoms associated with 6‐OHDA‐induced PD rats. Compared to previous key findings determined following the transplantation of iPSC‐derived progenitors or homogeneous iPSC‐derived dopaminergic neurons into rats with 6‐OHDA‐induced PD,^50^, ^51^, ^52^ motor symptom amelioration was initiated at a much earlier time point following the transplantation of neurons from the mini‐SNLSs (8 weeks vs at least 16 weeks post‐transplantation for previous attempts). The results of recent studies indicate that transplants of brain or other organ‐like structures are much more biofunctional than homogeneous populations of neurons or cells.^4^, ^53^ These comparisons clearly demonstrate the great potential of our mini‐SNLS for the achievement of better therapeutic results in subjects with PD.

The mini‐SNLSs appear to be very safe for potential clinical applications, given the following aspects: 1) the mini‐SNLSs self‐organized on iSECnMs comprising biocompatible silica nanostructures;^16^ 2) the lack of traditional GFs minimized undesirable effects in clinical applications; 3) mini‐SNLSs were derived from nongenetically manipulated NSCs and therefore confer a lower risk of tumorigenicity than iPSCs;^54^ and 4) the transplantation of mini‐SNLSs composed of mature and differentiated neurons allayed the risks of carcinogenicity and unwanted differentiation in vivo. Furthermore, mini‐SNLSs could be formed using neurons differentiated from autologous NSCs isolated from live subjects via our own nanotechnological methods, thus avoiding issues associated with ethics and immune rejection.^55^ The ability to engineer the physical cues of the iSECnMs flexibly by using GLAD to control the materials and nanostructures ensures that the iSECnMs will be generally adaptable. This enables the specific differentiation of stem cells to various lineage commitments, which could meet the current clinical demand for the development of effective cell therapies and ultimately treat incurable diseases.

## 4 Experimental Section

*GLAD of Silica iSECnMs*: SiO_2_ (99.99%, Kurt J. Lesker Company, PA, USA) was evaporated at a rate of ≈0.4 nm s^−1^ in a custom‐built physical vapor deposition system (JunSun Tech Co. Ltd., Taiwan) with a high vacuum of 10^−7^–10^−6^ Torr. This process was monitored by a quartz crystal microbalance located near the sample. An electron‐beam accelerating voltage of 8.0 kV and emission current of 83–87 mA were applied. Silica was deposited at a deposition angle (α) of 87° with respect to the substrate normal. The samples were deposited onto indium tin oxide (ITO)‐coated glasses (Xin Yan Technology Ltd.) and Si wafers (Semiconductor Wafer, Inc.). The substrate temperature was maintained at room temperature during GLAD using an ethanol/water cooling system. To produce left‐handed NHs, the supporting substrate was rotated counterclockwise at a rate *R*_r_ (in units of degree per second, or deg s^−1^) given by Equation (1)

(1) $$R_{r}\;=\;360\;R_{d}/P_{H}$$

where *P*_H_ is the helical pitch and *R*_d_ is the rate of silica deposition on the substrate surface, which was calibrated at 0.28 nm s^−1^ and an α of 87°. To produce NZs, the substrate was moved back and forth at 180° intervals, during which tilted nanorods were deposited at lengths (zigzag pitch or *P*_Z_) determined as a function of the duration of deposition. The iSECnM structures are summarized in Table S1 (Supporting Information). NSC differentiation was mediated on silica iSECnMs deposited on ITO‐coated glasses.

*Material Characterization*: The deposited samples were split mechanically such that the freshly exposed surfaces were available for structural characterization via SEM (Oxford, LEO 1530). The silica NHs and NZs were scratched from the substrates and fully dispersed in ethanol via ultrasonication for 5 min. Several drops of the mixture were then applied to a lacy carbon film on a grid structure (Electron Microscopy Sciences). The grid was dried under ambient conditions and inspected via transmission electron microscopy (TEM, Tecnai G2 20 STWIN). Samples not subjected to post‐GLAD treatment were characterized by X‐ray diffraction (XRD, Bruker, non‐monochromated Cu Kα X‐ray with a wavelength of 0.15418 nm, Advance D8 multipurpose X‐ray diffractometer), XPS (Sengyang SKL‐12, non‐monochromatic Mg Kα radiation of 1253.6 eV at a current of 15 mA, a voltage of 10 kV, a takeoff angle (between the sample and detector) of 90°, and a vacuum of ≈2 × 10^−9^ mbar), and nanoindentation (Ubi 1 Nanomechanical Test Instrument; three‐sided pyramidal tips with radii of 10–200 nm).

*NSC Isolation and Cell Culture*: The experimental protocol was approved by the Department of Health, the Government of the Hong Kong SAR, and was performed in accordance with the relevant guidelines and regulations of the Committee on the Use of Human and Animal Subjects in Teaching and Research (HASC) at HKBU. Rats (Sprague–Dawley, postnatal days 1–2) were purchased from the Chinese University of Hong Kong (CUHK). NSCs were dissected from the subventricular zone (SVZ) and cultured at an appropriate density in complete medium composed of neurobasal medium (Gibco 21 103, Thermo Scientific) supplemented with 10% FBS (Gibco 10 270, Thermo Scientific), 1% penicillin–streptomycin–neomycin (PSN; Gibco 15 640, Thermo Scientific), and 2% B27 supplement (Gibco 17 504, Thermo Scientific). Silica iSECnMs were sterilized in a steam autoclave at 121 °C for 20 min. NSCs were incubated on these sterilized structures without chemical modification at 37 °C in an environment containing 5% CO_2_.

*Live/Dead Assay*: The viability of cells cultured on the iSECnMs was measured using a live/dead viability/cytotoxicity kit (Invitrogen L3224, Thermo Scientific), according to the manufacturer's protocol. Viability is represented as the ratio of the numbers of living and total cells. A confocal microscope (FluoView FV1000, Olympus) was used to perform fluorescence imaging and differential interference contrast analyses. Quantitative analyses were conducted by manually counting at least six nonoverlapping areas per sample.

*Immunofluorescent Staining*: Prior to immunostaining, the cells were fixed with 4% paraformaldehyde (PFA, Sigma) for 30 min at room temperature and then incubated with specific dilutions of primary antibodies in phosphate‐buffered saline (PBS) containing 0.1% Triton X‐100 (Sigma) and 2% normal goat serum (Vector Laboratories) overnight at 4 °C. Next, the cells were stained with specific secondary antibodies for 3 h at room temperature. Finally, the cells were mounted with fluorescence mounting medium (Dako), and the immunoreactivity was imaged using a confocal microscope (FluoView FV1000, Olympus). The following primary antibodies were used in this study: anti‐Nestin (MAB353, Millipore, 1:500 dilution), anti‐TUJ1 (MAB1637, Millipore, 1:500), anti‐MAP2c (MAB364, Millipore, 1:500), anti‐GFAP (AB5804, Millipore, 1:500), anti‐GFP (ab6556, Abcam, 1:500), anti‐TH (AB152, Millipore, 1:500), anti‐GAD (AB5992, Chemicon, 1:500), anti‐VGLUT2 (AB2251, Millipore, 1:500), and antioligodendrocytes (Oligo; MAB1580, Millipore, 1:500).

*Western Blotting Assay*: Western blotting assays were used to compare the levels of cellular proteins that were extracted using a protein extraction reagent (Novagen) supplemented with a protease inhibitor cocktail (Calbiochem). An aliquot of each sample containing 30 µg of total protein was separated on a 10% sodium dodecyl sulfate (SDS)–polyacrylamide gel and transferred to a polyvinylidene difluoride (PVDF, Bio‐Rad) membrane. The membrane was probed with various primary antibodies overnight at 4 °C and subsequently incubated with complementary secondary antibodies at room temperature for 1 h. A β‐actin antibody (A5316, Sigma, 1:5000) was used as a protein loading reference. Images of the labeled bands were captured using a ChemiDoc Touch imaging system (Bio‐Rad). The following primary antibodies were used in this study: anti‐Nestin (1:1000 dilution), anti‐TUJ1 (1:1000), anti‐MAP2c (1:1000), anti‐GFAP (1:1000), anti‐p‐GSK‐3β (Ser9) (9336S, Cell Signaling, 1:1000), anti‐GSK‐3β (9315, Cell Signaling, 1:1000), anti‐non‐p (Active) β‐catenin (8814S, Cell Signaling, 1:1000), anti‐β‐catenin (8480P, Cell Signaling, 1:1000), anti‐integrin β1 (MAB1997, Millipore, 1:1000), anti‐p‐RhoA (S188) (ab125275, Abcam, 1:1000), anti‐RhoA (2117S, Cell Signaling, 1:1000), anti‐Myosin IIB (ab24761, Abcam, 1:1000), anti‐p‐p44/42 MAPK(Erk1/2) (Thr202/Tyr204) (4370S, Cell Signaling, 1:1000), anti‐p44/42 MAPK (Erk1/2) (4695S, Cell Signaling, 1:1000), anti‐GLI1 (3538S, Cell Signaling, 1:1000), anti‐TH (1:1000), anti‐GAD (1:1000), anti‐VGLUT2(1:1000), and anti‐Oligo (1:1000).

*AFM Characterization of Differentiated NSCs*: AFM nanoindentation (Bioscope Catalyst AFM, Bruker) was used to study the mechanical properties of cells differentiated on different substrates. This experiment used a specific AFM probe (MSCL‐D probe, Bruker) with a spring constant that had been calibrated experimentally (≈0.05 N m^−1^) using the thermal tune method. The Young's modulus (*E*) of a cell was evaluated by fitting the force–indentation curves using the Sneddon model, given by Equation (2)

(2) $$F\;=\;2E\delta^{2}\tan\beta /\left[\pi \left(1-\nu^{2}\right)\right]$$

where *F* is the loading force, ν is the Poisson ratio of a cell (0.5), β is the half‐angle of an AFM tip (18°), and δ is the indentation depth. Approximately 30 cells were investigated per differentiation substrate, and 64 force–distance curves were monitored at the centers of individual cells to enable a statistical evaluation of the Young's modulus of the differentiated cells.

The PeakForce Quantitative Nanomechanical Mapping (QNM) mode and OLTESPA probe (Bruker) were used to image the cellular topography. Differentiated cells were fixed with 4% PFA in Hank's balanced salt solution (HBSS, ThermoFisher) for 30 min at room temperature prior to AFM imaging. The loading force was controlled at a maximum of 8 nN to moderate cell deformation. Cells were imaged on the NSC differentiation substrates in HBSS. The offline analysis was performed using NanoScope analysis.

*Electrophysiological Characterization*: The patch clamp technique was used to record the ion channel activities of living cells. The whole‐cell voltage clamp was used to monitor the total current flow across the entire membrane of a cell due to the total ion channel activity in response to voltage stimuli. Control and differentiated cells were bathed in an extracellular solution inside a Petri dish, which was then placed on an inverted microscope (Eclipse Ti, Nikon). The extracellular solution contained 160 × 10^−3^ m NaCl, 4.5 × 10^−3^ m KCl, 1 × 10^−3^ m MgCl_2_, 2 × 10^−3^ m CaCl_2_, 5 × 10^−3^ m glucose, and 10 × 10^−3^ m 4‐(2‐hydroxyethyl)‐1‐piperazineethanesulfonic acid (HEPES), and the pH was adjusted to 7.4 using NaOH. The inward and outward currents of individual cells were recorded in the whole‐cell voltage‐clamp configuration using an Axopatch 200B patch clamp amplifier, Digidata 1440A data digitalizer, and pClamp 10.7 software (all from Molecular Devices, Sunnyvale, CA, USA). The data were sampled at 50 kHz, with a 5 kHz low‐pass filter. The electrode comprised a glass micropipette filled with an intracellular solution of 75 × 10^−3^ m KCl, 10 × 10^−3^ m NaCl, 70 × 10^−3^ m KF, 2 × 10^−3^ m MgCl_2_, 10 × 10^−3^ m HEPES, and 10 × 10^−3^ m ethylene glycol‐bis(2‐aminoethyl ether)‐*N*,*N*,*N*′,*N*′‐tetraacetic acid (EGTA) with a pH of 7.2–7.4 (adjusted with KOH). Twelve differentiated cells per group were selected for the statistical evaluation of the electrophysiological properties. To characterize the electrophysiological properties of dopaminergic neurons, the external solution was modified to contain 150 × 10^−3^ m NaCl, 5 × 10^−3^ m KCl, 1 × 10^−3^ m MgCl_2_, 2 × 10^−3^ m CaCl_2_, 10 × 10^−3^ m glucose, and 10 × 10^−3^ m HEPES with a pH 7.4, and the pipette solution contained 107 × 10^−3^ m KCl, 1.2 × 10^−3^ m MgCl_2_, 1.0 × 10^−3^ m CaCl_2_, 10 × 10^−3^ m EGTA, 5.0 × 10^−3^ m HEPES, and 3.0 × 10^−3^ m MgATP with a pH of 7.3. Four differentiated cells were selected per group.

*In Vivo Survival of the Differentiated Cells from NSCs Induced by Silica iSECnMs*: NSCs were differentiated on silica NZs *^P^* ^225^ for 4, 7, and 14 days. Subsequently, the cells were transfected with Lenti‐GFP (GeneCopoeia) at a multiplicity of infection (MOI) of 5 and monitored using fluorescence microscopy. Adult rats were purchased from CUHK and anesthetized with pentobarbital prior to the experiments. All animal experimental procedures were approved by the Department of Health, HKSAR Government and the HASC at HKBU. Single GFP^+^ cell solutions were prepared and stored on ice prior transplantation. A total of 2 × 10^5^ cells suspended in PBS (4 × 10^4^ cells µL^−1^) were unilaterally injected into the right parietal cortex of each rat via a Hamilton microsyringe (Hamilton Company) at a rate of ≈1 µL min^−1^. Seven days postinjection, the transplanted rats were anesthetized again and perfused. Brain tissues were harvested directly and fixed first in 4% PFA overnight at room temperature and then in 0.9% normal saline for 2 days. Coronal sections (50 µm) of the postfixed brains were sliced using a freezing‐stage sledge microtome. To determine the viability of the differentiated neurons in vivo, brain sections were subjected to immunofluorescence staining to visualize the protein markers GFP, TUJ1, GAD, VGLUT2, and Oligo.

*Therapeutic Effect of the Self‐Organized Mini‐SNLS in a Rat Model of 6‐OHDA‐Lesioned PD*: The experimental procedure was approved by the Department of Health, HKSAR Government and the HASC of HKBU. Adult male rats (body weight: 260–300 g) were anaesthetized with ketamine (100 mg kg^−1^) and xylazine (10 mg kg^−1^). To establish a nigro‐striatal pathway PD model, unilateral medial forebrain bundle (MFB) lesions were made by a single stereotaxic injection of 6‐OHDA (4 mg mL^−1^ in 0.1% ascorbic acid and 0.9% saline; H4381, Sigma) at the following brain coordinates: anteroposterior (AP) −2.2 mm, mediolateral (ML) 1.5 mm, and dorsoventral (DV) −8.0 mm. At 4–5 weeks postinjection, rats that exhibited apomorphine (A4393, Sigma)‐induced contralateral rotations at a rate exceeding 7 rpm within 30 min were selected as either control rats (no transplantation; *n* = 6) or for transplantation with self‐organized mini‐SNLSs (mini‐SNLS rats; *n* = 6). In the latter group, 2 × 10^5^ cells differentiated from NSCs on silica NZs *^P^* ^225^ for 14 days were labeled with GFP and transplanted into the striatum (coordinates: AP 1.0 mm, ML 2.5 mm, and DV −4.7 mm) of each mini‐SNLS rat. Apomorphine‐induced rotations were monitored during the second week before mini‐SNLS transplantation and the 4th, 8th, 12th, and 18th weeks post‐transplantation, after which the rats were perfused for histological analysis (Figure 4a). Coronal brain sections (7 µm) were subjected to immunofluorescence staining (visualization of GFP, TH, and 4′,6‐diamidino‐2‐phenylindole (DAPI)) and H&E staining, respectively.

*Statistical Analysis*: At least three individual experiments were performed, and the means ± standard deviations (s.d.) were calculated for all data, except AFM and electrophysiological data for which the means ± standard errors of the means (s.e.m.) were calculated. Statistical significance was determined using a one‐way analysis of variance (ANOVA) and defined as a *p* value of <0.05. ImageJ (National Institutes of Health, Bethesda, MD, USA) was used for image and cell counting analyses. All graphs were produced using Prism 5.0 (GraphPad Inc., La Jolla, CA, USA).

## Conflict of Interest

The authors declare no conflict of interest.

## Supporting information

Supplementary
