# Supplementary material for: Extracellular Nanomatrix‐Induced Self‐Organization of Neural Stem Cells into Miniature Substantia Nigra‐Like Structures with Therapeutic Effects on Parkinsonian Rats
Source: Adv Sci (Weinh). 2019 Sep 30;6(24):1901822. doi: 10.1002/advs.201901822 (PMC6918115; doi:10.1002/advs.201901822)
Supplement: Supplementary file 1 — Supplementary [file ADVS-6-1901822-s001.pdf]

Copyright WILEY-VCH Verlag GmbH & Co. KGaA, 69469 Weinheim, Germany, 2019.

ADVANCED SCIENCE Open Access

Supporting Information

for *Adv. Sci.,* DOI: 10.1002/advs.201901822

Extracellular Nanomatrix-Induced Self-Organization of Neural Stem Cells into Miniature Substantia Nigra-Like Structures with Therapeutic Effects on Parkinsonian Rats

Shiqing Zhang, Peng Sun, Kaili Lin, Florence Hiu Ling Chan, Qi Gao, Wai Fung Lau, Vellaisamy A. L. Roy, Hongqi Zhang, King Wai Chiu Lai, Zhifeng Huang,* and Ken Kin Lam Yung**

Copyright WILEY-VCH Verlag GmbH & Co. KGaA, 69469 Weinheim, Germany, 2019.

Supporting Information

Extracellular Nanomatrix-Induced Self-Organization of Neural Stem Cells into Miniature Substantia Nigra-Like Structures with Therapeutic Effects on Parkinsonian Rats

Shiqing Zhang,[#] Peng Sun,[#] Kaili Lin,[#] Florence Hiu Ling Chan, Qi Gao, Waifung Lau, Vellaisamy A. L. Roy, Hongqi Zhang, King Wai Chiu Lai, Zhifeng Huang,* and Ken Kin Lam Yung**

Dr. S. Q. Zhang, K. L. Lin, Prof. K. K. L. Yung
Department of Biology, Hong Kong Baptist University (HKBU), Kowloon Tong, Kowloon, Hong Kong SAR, China.
E-mail: kklyung@hkbu.edu.hk

P. Sun, W.F. Lau, Prof. Z. F. Huang
Department of Physics, HKBU, Kowloon Tong, Kowloon, Hong Kong SAR, China.
E-mail: zfhuang@hkbu.edu.hk

F. H. L. Chan, Q. Gao, Prof. K. W. C. Lai
Department of Biomedical Engineering, City University of Hong Kong (CityU), Tat Chee Avenue, Kowloon Tong, Kowloon, Hong Kong SAR, China.
E-mail: kinglai@cityu.edu.hk

Dr. V. A. L. Roy
Department of Materials Science and Engineering, CityU, Tat Chee Avenue, Kowloon Tong, Kowloon, Hong Kong SAR, China.

Prof. H. Q. Zhang
School of Chinese Medicine, HKBU, Kowloon Tong, Kowloon, Hong Kong SAR, China.

Dr. S. Q. Zhang, K. L. Lin, Dr. Z. F. Huang, Prof. K. K. L. Yung

Golden Meditech Center for NeuroRegeneration Sciences, Hong Kong Baptist University, Kowloon Tong, Kowloon, Hong Kong SAR, China.

Dr. S. Q. Zhang, Dr. Z. F. Huang, Prof. K. K. L. Yung
HKBU Institute of Research and Continuing Education, 9F, the Industrialization Complex of Shenzhen Virtual University Park, No. 2 Yuexing 3rd Road, South Zone, Hi-tech Industrial Park, Nanshan District, Shenzhen, 518057, Guangdong Province, China.

P. Sun
Department of Materials Science and Engineering, Southern University of Science and Technology, Shenzhen, Guangdong 518000, China

Dr. Z. F. Huang, Prof. K. K. L. Yung
Institute of Advanced Materials, State Key Laboratory of Environmental and Biological Analysis, HKBU, Kowloon Tong, Kowloon, Hong Kong SAR, China.

[#]S.Q.Z., P.S. and K.L.L. contributed equally to this work.

Table S1. Summary of the sculptured structures and stiffness (Young's modulus) of silica and TiO_x inorganic sculptured extracellular nanomatrices (iSECnMs). *H*: height, *P*: helical or zigzag pitch, *n*: number of pitches, *d*: wire diameter. To statistically evaluate *H* and *d*, each sample was measured multiple (at least 10) times, and the algebraic average value ± standard deviation (s.d.) was evaluated. The contact depths (d_c) of the differentiated cells were calculated as $d_c=(P+d)/2$ for nanohelices (NHs), and $d_c=P+d/2$ for nanozigzags (NZs). To evaluate the Young's modulus values using nanoindentation, iSECnMs were deposited on a silicon wafer, and six points over the wafer surface of each sample were measured for the statistical evaluation.

iSECnMs		*H* (nm)	*n*	*d* (nm)	*P = (H-d)/n* (nm)	d_c (nm)	Young's modulus (GPa)
Silica	NHs	538 ± 4	2	48 ± 2	245 ± 4	146 ± 4	3.20 ± 0.24
	NZs^{P80}	279 ± 7	3	38 ± 2	80 ± 7	99 ± 7	0.62 ± 0.08
	NZs^{P170}	553 ± 3	3	46 ± 3	169 ± 4	192 ± 4	2.04 ± 0.22
	NZs^{P225}	733 ± 7	3	64 ± 4	223 ± 8	255 ± 8	7.85 ± 1.39
TiO_x	NHs	480 ± 3	2	47 ± 3	217 ± 4	132 ± 5	0.63 ± 0.09
	NZs^{P110}	365 ± 7	3	42 ± 6	108 ± 9	129 ± 9	0.62 ± 0.04

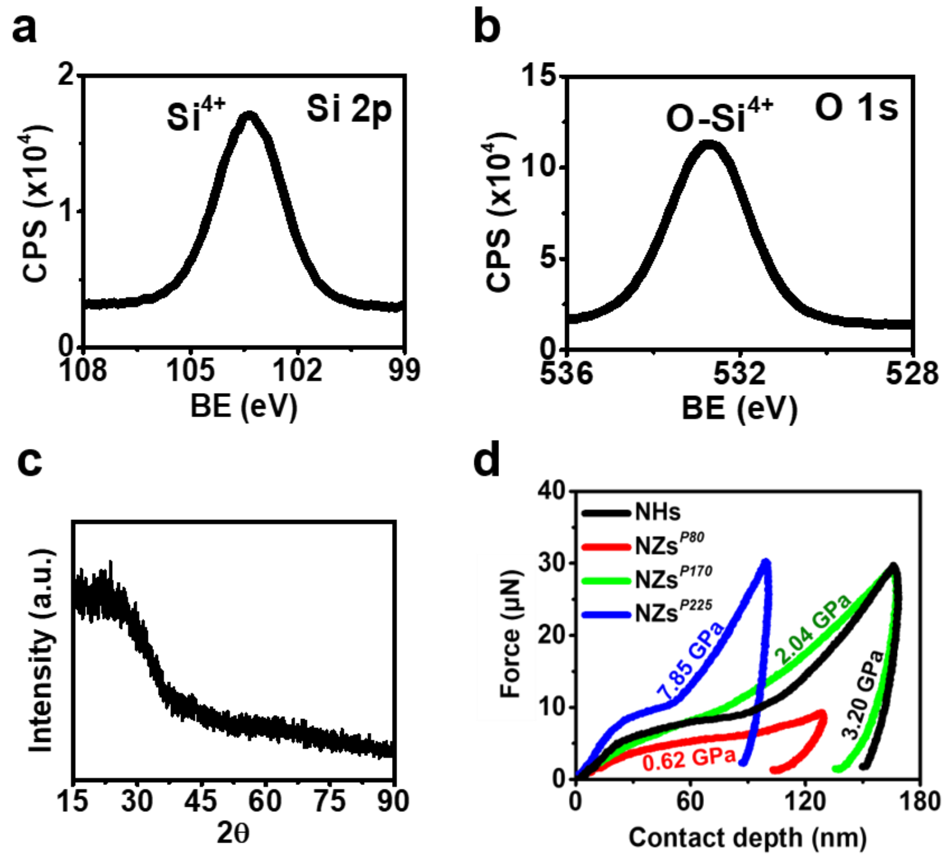

Figure S1. Characterizations of the silica inorganic sculptured extracellular nanomatrices (iSECnMs). a, b) X-ray photoelectron spectra of the silica nanohelices (NHs): Si2p (a), O1s (b). c) X-ray diffraction spectrum of the silica NHs deposited on glass. d) Nanoindentation measurements of the Young's modules of the silica NHs (black line) and nanozigzags (NZs) with diverse P_z values (NZs^{P80}: red line, NZs^{P170}: green line, and NZs^{P225}: blue line). The numbers in the plots represent the algebraic averages of Young's modules.

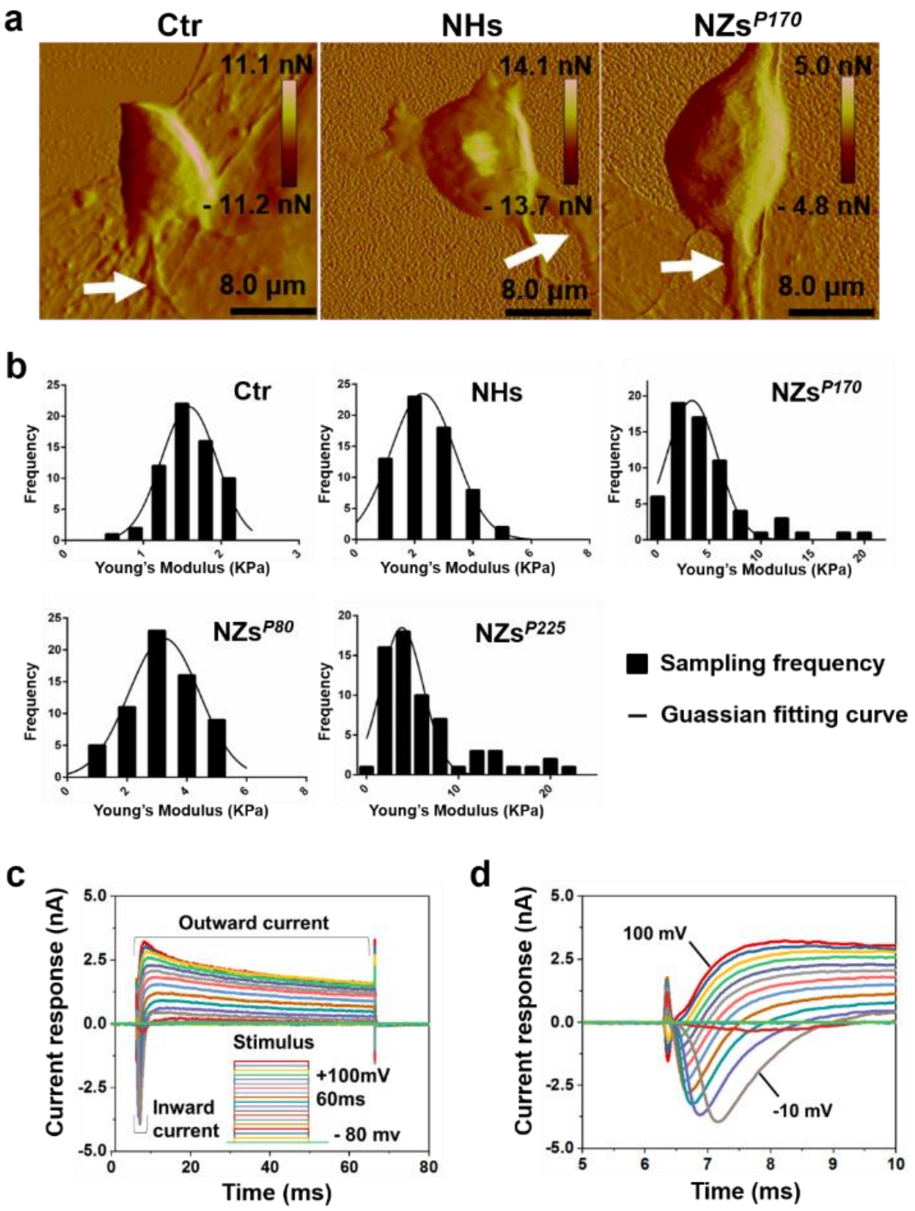

Figure S2. Mechanical and electrophysiological characterizations of the neuronal cells differentiated on silica inorganic sculptured extracellular nanomatrices (iSECnMs). a) Atomic force microscopy peak force error images of differentiated neuronal cells on control glass (control group, Ctr), nanohelices (NHs), and nanozigzags with a P_z value of 170 nm (NZs^{P170}). Axon-like structures are marked by white arrows. b) Histograms of the Young's modulus of differentiated neuronal cells in the control group (Ctr) and those differentiated on silica iSECnMs sculptured into NHs, NZs^{P80}, NZs^{P170}, and NZs^{P225}. The solid black curves represent the Gaussian fitting of the histograms. c, d)

Inward and outward currents of the differentiated neurons on the NZs^{P170}, ranging from 0 to 70 ms (c) and from 5 to 10 ms (d).

S3. Neural stem cell (NSC) differentiation mediated by TiO_x inorganic sculptured extracellular nanomatrices (iSECnMs).

The iSECnMs were sculpted from titanium oxides (TiO_x) into left-handed nanohelices NHs (Figures S3a and S3c) and nanozigzags (NZs; Figures S3b and S3d) using glancing angle deposition (GLAD). The nanostructures are summarized in Table S1. An X-ray photoelectron spectrum depicts Ti^{3+} and Ti^{4+} ions at the iSECnM surfaces (Figures S3e and S3f), indicating $1.5 \leq x \leq 2$. An X-ray diffraction (XRD) characterization yielded $0.33 \leq x \leq 1.5$ in the cores (Figure S3g). The inability of XRD to detect TiO_2 on the nanostructure surfaces is probably due to the excessive thinness of the surface layers of TiO_2. Here, the deposited TiO_x is depicted as a core with $0.33 \leq x \leq 1.5$ and shells with $1.5 \leq x \leq 2$, indicating that spontaneous surface oxidation prohibits further core oxidation. The TiO_x iSECnMs formed crystalline structures (Figure S3g). The Young's modules of the TiO_x iSECnMs were approximately 0.62 GPa, and this value was independent of the sculptured shape (Figure S3h).

Regarding *in vitro* NSC differentiation, the TiO_x iSECnMs suppressed the expression of Nestin (day 4), TUJ1 (day 7), and MAP2c (day 7) proteins relative to the levels in the control group, but enhanced the expression of GFAP. The TiO_x iSECnMs preferentially induced the astrocyte lineage over the neuronal lineage (Figure S4a). Additionally, the TiO_x iSECnMs tended not to increase the ratio of p-RhoA (S188) to total RhoA or to suppress the expression of Myosin IIB, but did upregulate the expression of integrin β1 slightly (Figure S4b).

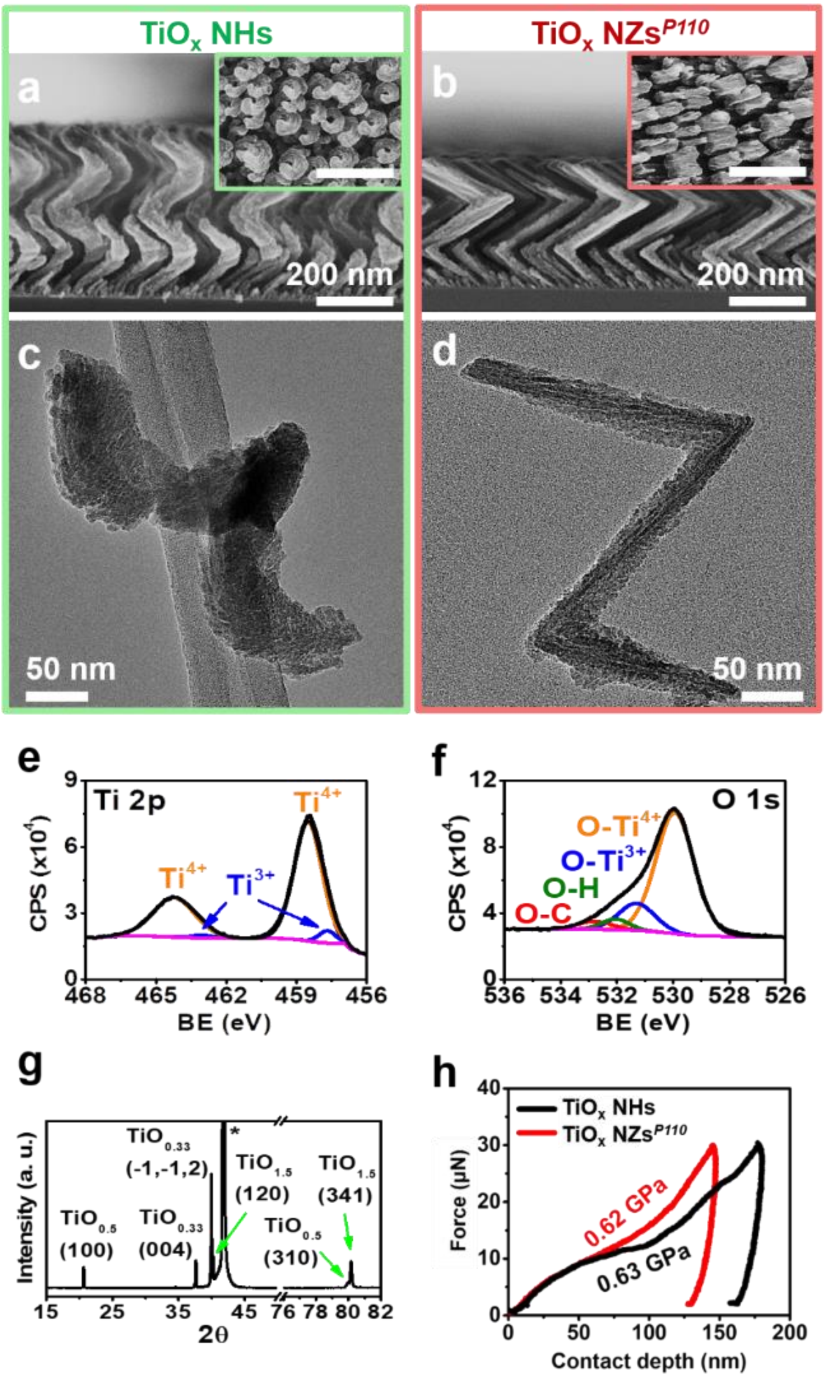

Figure S3. Glancing angle deposition (GLAD) and characterizations of the TiO_x inorganic sculptured extracellular nanomatrices (iSECnMs) sculptured into nanohelices (NHs) (a, c) and nanozigzags with a P_z of 110 nm (NZs^{P110}) (b, d). The structures of the TiO_x NHs and NZs^{P110} are summarized in Table S1. a, b) Cross-sectional scanning electron microscopy (SEM) images of the samples (insets: SEM top-down images). Scale bars: 200 nm. c, d) Transmission electron

microscopy (TEM) images of individual nanostructures. Scale bars: 50 nm. e, f) X-ray photoelectron spectra of TiO_x NZs^{P110} deposited on a sapphire: (e) Ti2p, and (f) O1s. g) X-ray diffraction spectra of TiO_x NZs^{P110} deposited on a sapphire; and the peak marked by an asterisk is assigned to the sapphire. h) Nanoindentation measurement of the Young's modules of the samples (NHs: black line; NZs: red line). The numbers shown in the plots represent the algebraic averages of Young's modules.

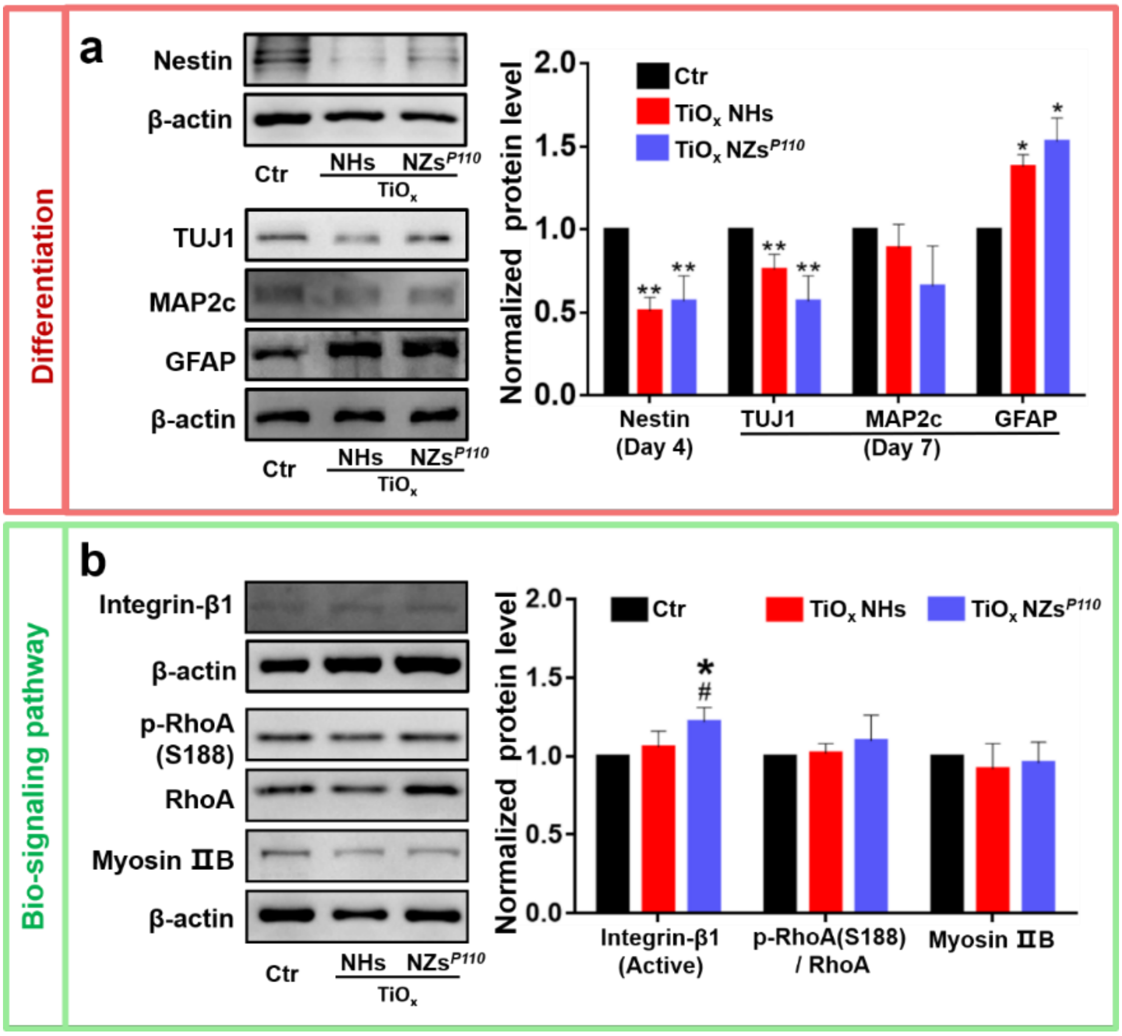

Figure S4. Differentiation of neural stem cells (NSCs) on the TiO_x inorganic sculptured extracellular nanomatrices (iSECnMs) sculptured into nanohelices (NHs) and nanozigzags with a P_z of 110 nm (NZs^{P110}). a) NSCs differentiated on TiO_x iSECnMs were characterized by western blotting of the expression of various protein markers on day 4 (Nestin) and day 7 (TUJ1, MAP2c, and GFAP). b) Western blot analyses and relative optical densities of various markers in the integrin β1/RhoA/Myosin IIB pathway, including integrin β1, p-RhoA(S188), RhoA, and Myosin IIB. The relative optical densities of these proteins were assessed using β-actin as a reference. Data are shown as means ± s.d.; *$p < 0.05$ and **$p < 0.01$, compared with the control group; $^{\#}p < 0.05$, compared with TiO_x NHs^{P110}.

S4. Signaling pathways activated by silica iSECnMs and the associated biological network.

The physical cues of silica iSECnMs activate several multiple signaling pathways to induce NSC differentiation and enable the self-organization of mini-SNLSs(Figure 3e). Integrin β1 is a heterodimeric surface molecule that regulates intracellular and extracellular signaling pathways to influence stem cell survival, migration, and differentiation.[S1] RhoA plays a crucial role in regulating cellular responses to mechanical cues. The inactivation of RhoA via phosphorylation at Ser188 [p-RhoA (S188)], as well as inactivation of the downstream factor Myosin IIB, can promote the differentiation of NSCs into dopaminergic neurons[S2] (marked in green). Wnt/β-catenin signaling is thought to be a critical regulator of NSC induction.[S3] Inactivation of GSK-3β by phosphorylation at Ser9 [p-GSK-3β (Ser9)] can induce the accumulation of active β-catenin in the cytoplasm and activate the Wnt/β-catenin pathway (marked in pink). The Erk1/2 pathway, which is activated via phosphorylation at Thr202/Tyr204, contributes to the differentiation of NSCs[S4] (marked in blue). GLI1 is a crucial transcription factor and activation marker in the SHH pathway[S5] (marked in red). Increases in the expression of integrin β1 (Figure 3f); the ratios of p-RhoA (S188) to total RhoA (Figure 3f), p-GSK-3β (Ser9) to total GSK-3β, and active β-catenin to total β-catenin (Figure 3g); the activation of Erk1/2 (Figure 3h), and the expression of GLI1 (Figure 3i) were observed in the following order from weakest to strongest: control glass, the NHs and the NZs^{P170}. Suppression of Myosin IIB expression was also observed (Figure 3f).

Notably, RhoA is a central effector of cross-talk and tends to interact with other signaling pathways. For example, RhoA inactivation can enhance Wnt/β-catenin signaling,[S6] while RhoA inhibition leads to Erk activation,[S7] and vice versa.[S8] GLI1 overexpression has been observed in the RhoA-deficient midbrain,[S9] and is activated in response to Erk1/2 activation[S10]. However, mediation by iSECnMs did not induce significant changes in the ratio of p-Akt (Ser473) to total Akt expression (Figure S5), indicating that the Akt pathway is not involved in iSECnM-mediated NSC

differentiation. This pathway is another regulator of NSC differentiation and is activated via phosphorylation at Ser473 [p-Akt (Ser473)].[S11]

These results strongly emphasize that silica iSECnMs, especially NZs^{P170}, effectively activated the integrin β1/RhoA/Myosin IIB pathway, which interacts with multiple other signaling pathways. This observation is consistent with the results reported from previous studies of nerve GF-induced NSC differentiation via RhoA inactivation or Erk activation. [S12, S13] In conventional SHH-induced dopaminergic and GABAergic neuronal differentiation, SHH binds the transmembrane protein Ptch1 and consequently upregulates GLI1 as a transcription factor associated with the SHH pathway.[S5] The activation of additional genes effectively induces dopaminergic and GABAergic neuronal differentiation.

Our findings show that the physical cues of NZs^{P170} induce the upregulation of integrin β1, suppress the activation of RhoA, promote activation of the Wnt/β-catenin and Erk1/2 pathways, and eventually induce the upregulation of GLI1 via this biological network. Silica NZs therefore enhance GLI1 expression directly and mimic the GF effect of SHH to promote the differentiation of dopaminergic and GABAergic neurons.

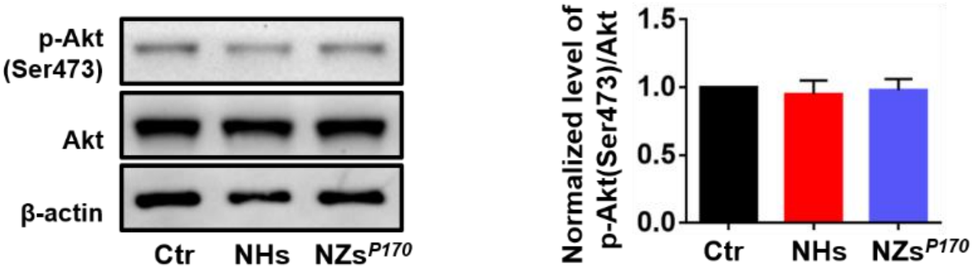

Figure S5. The activation of Akt in neural stem cells (NSCs) differentiated on silica inorganic sculptured extracellular nanomatrices (iSECnMs). Western blot analyses and relative optical densities of the Akt pathway markers p-Akt (Ser473) and Akt. β-actin was used as a reference protein. Data are shown as means ± s.d.

S5. *In vivo* survival of the differentiated mini-SNLS.

The survival of NSCs differentiated on silica NZs^{P225} for 4, 7, and 14 days was analyzed after the cells were labeled with GFP and transplanted into the parietal cortices of adult rats. Immunofluorescence in the cerebral sections was analyzed on day 7 post-transplantation (Figure S6a). An increased period of differentiation on the NZs ^{P225} evidently induced GFP^{+} and TUJ1^{+} cells to disseminate widely around the primary transplantation site (Figures S6b-e). After a 14-day period of differentiation, the differentiated neuronal cells exhibited neurite-like and neuronal perikaryal structures. GFP^{+} and TUJ1^{+} cells with bouton-like structures were observed in close proximity to the neuronal perikaryals (Figure S6e). Additionally, although some TH^{+} and GAD^{+} cells were visible around the primary transplantation site, VGLUT2^{+} or Oligo^{+} cells were rarely observed (Figure S6f). Some GFP^{+} cells migrated towards the top of the cortex (Figure S7a), and many GFP^{+} and TUJ1^{+} cells exhibiting neurite-like structures appeared to distribute widely from the primary transplantation site (Figures S7b–d). These findings confirmed the migration of the differentiated neuronal cells and the extension of neurites into the transplanted cortex. The silica iSECnMs tended to correlate with NSC differentiation and the subsequent survival of the transplanted cells in the absence of neurogenic chemical cues. After a 1-week culture of NSCs on silica NZs ^{P225}, most of the GFP^{+} cells remained immaturely differentiated. However, after a 2-week differentiation, many transplanted cells exhibited a mature pattern of neuronal differentiation and GFP^{+} bouton-like structures that were likely neuronal synapses. These findings indicate a 2-week NZ-mediated culture yielded maturely differentiated NSCs that could form functional synaptic connections with the neurons in the brain *in situ*. These results were superior to those reported from a previous study wherein the NSCs survived poorly after direct transplantation in the cerebral cortex.[S14]

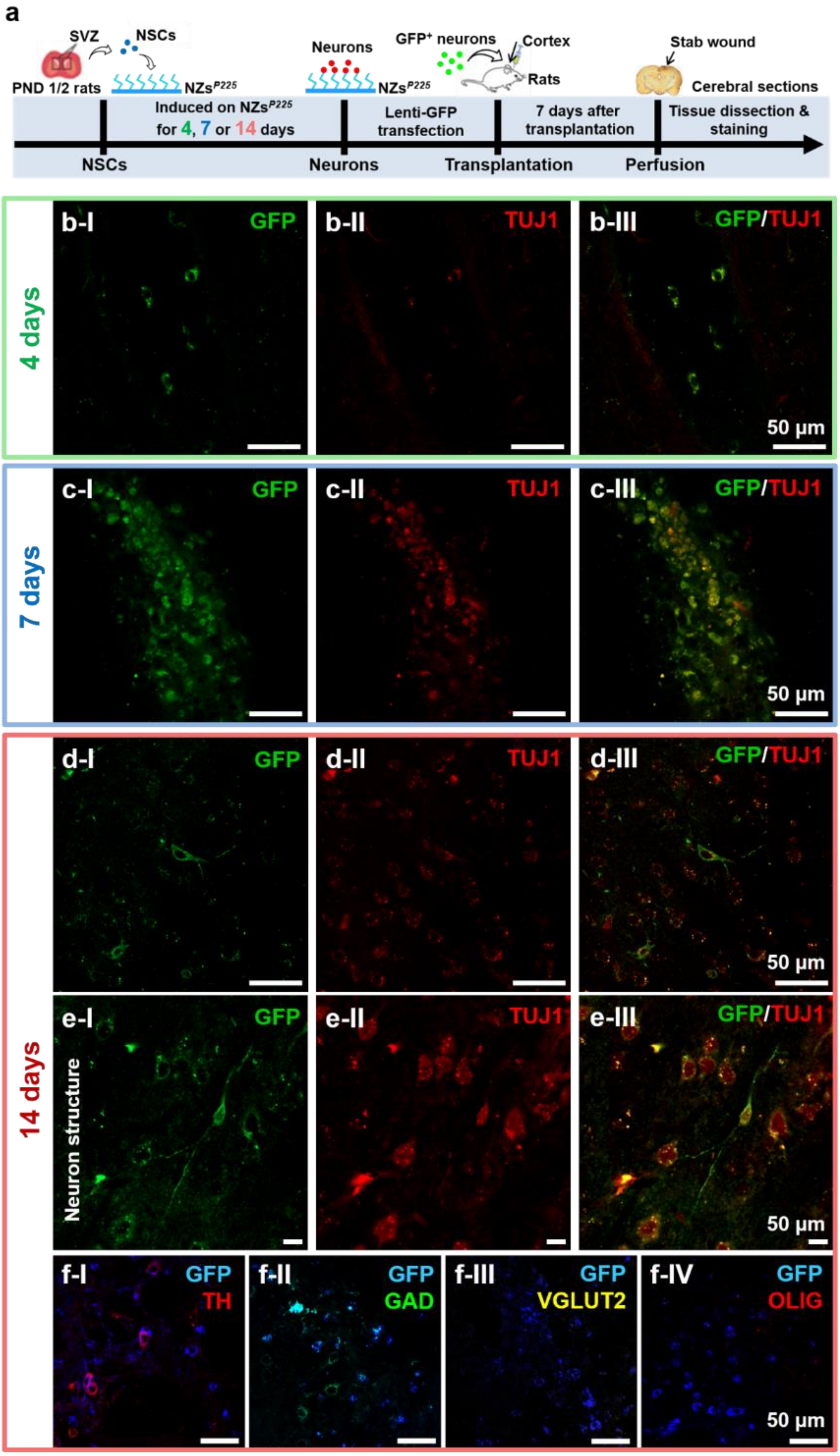
a
SVZ
NSCs
PND 1/2 rats
NZs^{P225}
Induced on NZs^{P225} for 4, 7 or 14 days
Neurons
NZs^{P225}
Lenti-GFP transfection
GFP^{+} neurons
Cortex
Rats
7 days after transplantation
Stab wound
Cerebral sections
Tissue dissection & staining
NSCs
Neurons
Transplantation
Perfusion
4 days
b-I GFP
b-II TUJ1
b-III GFP/TUJ1
50 μm
7 days
c-I GFP
c-II TUJ1
c-III GFP/TUJ1
50 μm
14 days
d-I GFP
d-II TUJ1
d-III GFP/TUJ1
50 μm
Neuron structure
e-I GFP
e-II TUJ1
e-III GFP/TUJ1
50 μm
f-I GFP TH
f-II GFP GAD
f-III GFP VGLUT2
f-IV GFP OLIG
50 μm

Figure S6. Transfection and transplantation of differentiated neurons into the parietal cortices of adult rats. a) An overview of the protocols of neuron transfection and transplantation. Neural cells were induced on silica nanozigzags (NZs) with a P_Z of ~225 nm (NZs^{P225}) for 4 (b), 7 (c), or 14 days (d, e, f) prior to transplantation into the adult cortex. b-d) Immunofluorescent images were obtained in the vicinity of the primary transplantation site on day 7 post-transplantation: (I) immunolabeled GFP$^+$ transplanted cells (in green), (II) TUJ1$^+$ cells (in red), and (III) a merged image of (I) and (II). e) Immunofluorescent images of transplanted neurons in the adult cortex: (e-I) GFP (in green), (e-II) TUJ1 (in red), and (e-III) a merged image of (e-I) and (e-II). f) Immunofluorescent images of transplanted cells in the adult cortex labeled specific neuron markers: (f-I) merged image of TH (in red) and GFP staining (in blue), (f-II) merged image of GAD (in green) and GFP staining (in blue), (f-III) merged image of VGLUT2 (in yellow) and GFP staining (in blue), and (f-IV) merged image of OLIG (in red) and GFP staining (in blue). Scale bars: 50 μm.

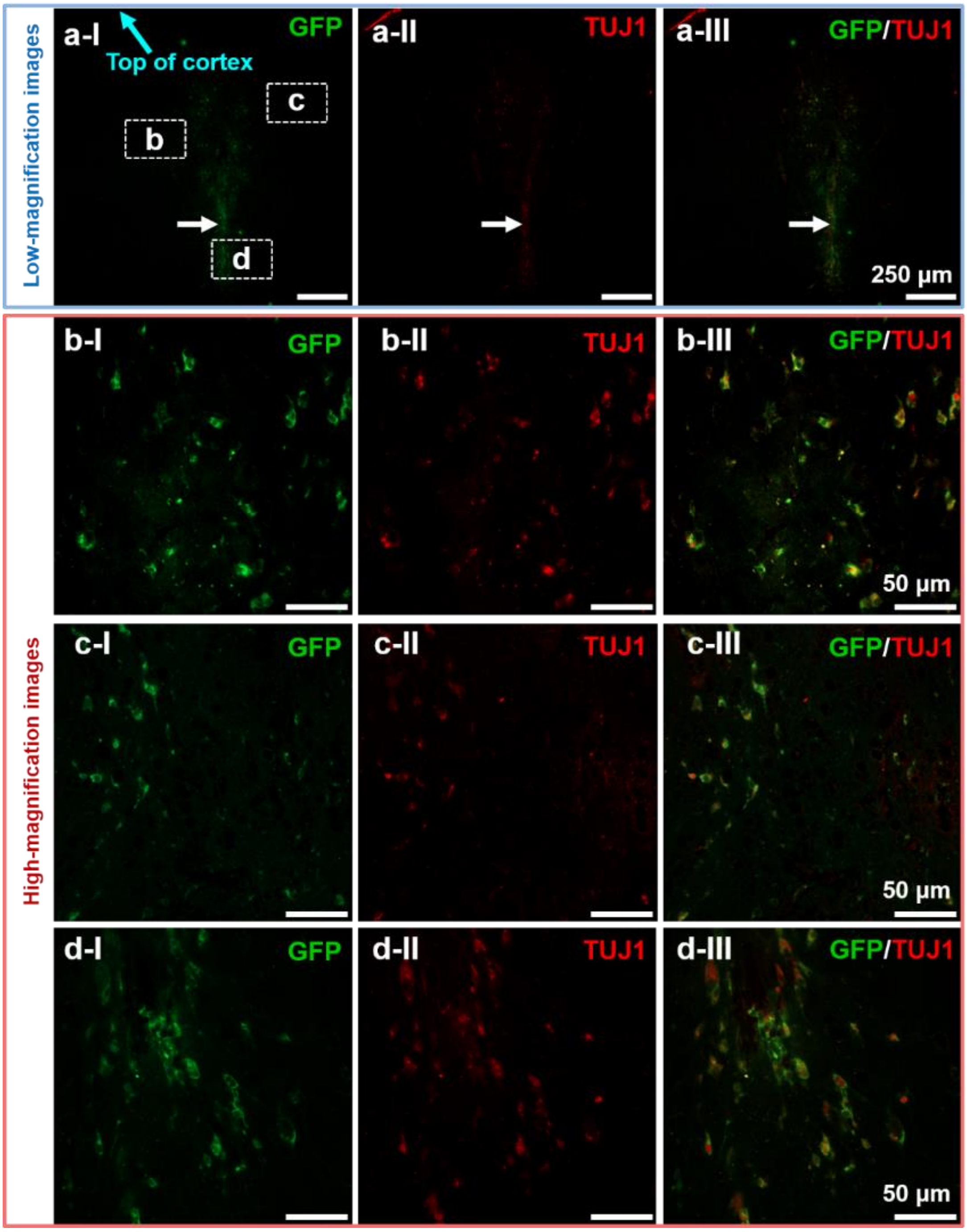

Figure S7. Valid migration of differentiated neurons after transplantation into the parietal cortex of an adult rat. Neural cells were differentiated on silica nanozigzags (NZs) with a P_Z of ~225 nm (NZs^{P225}) for 14 days before transplantation into an adult cortex. a) Low-magnification images were obtained near the primary transplantation site (marked by white arrows) on day 7 post-transplantation. b-d) High-magnification images depict three regions in the vicinity of the primary transplanted site, which are marked in a-I. I, GFP staining (in green); II, GFP plus TUJ1 immunolabeling (in red); III, merged image of I and II. Scale bars: 250 μm in a; 50 μm in b, c, d.

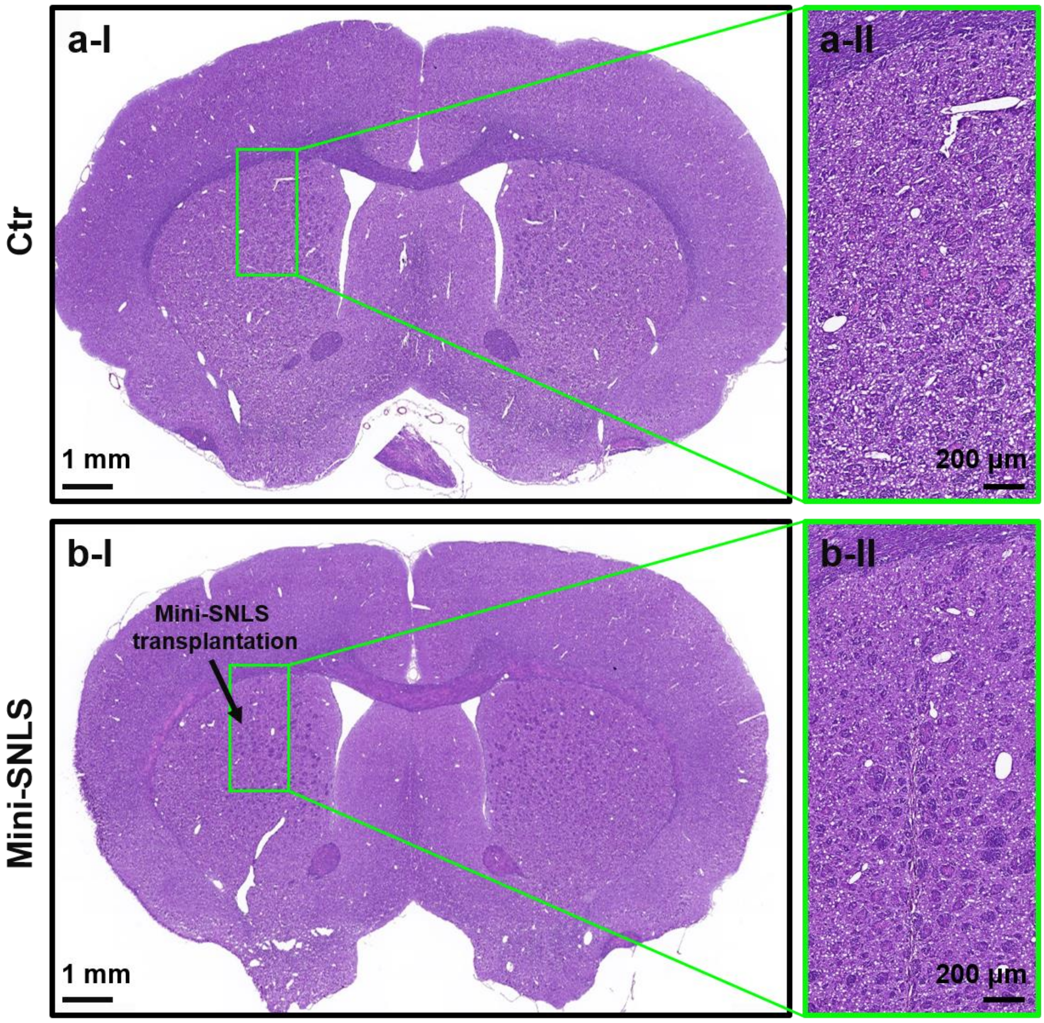

Figure S8. Hematoxylin and eosin (H&E) staining on brain coronal sections from (a) Ctr rat and (b) mini-SNLS rat in the eighteenth week post-transplantation. The box in each image outlines the area magnified to the right. Black arrow indicated the transplantation site of mini-SNLS. Scale bar: 1 mm in a-I, b-I; 200 μm in a-II, b-II.

Supporting References

[S1]J. Du, X. F. Chen, X. D. Liang, G. Y. Zhang, J. Xu, L. R. He, Q. Y. Zhan, X. Q. Feng, S. Chien, C. Yang, *Proc. Natl. Acad. Sci. U. S. A.* **2011**, *108*, 9466.

[S2]D. S. Wheeler, S. M. Underhill, D. B. Stolz, G. H. Murdoch, E. Thiels, G. Romero, S. G. Amara, *Proc. Natl. Acad. Sci. U. S. A.* **2015**, *112*, E7138.

[S3]M. Joksimovic, R. Awatramani, *J. Mol. Cell Biol.* **2014**, *6*, 27.

[S4]W. S. Chan, A. Sideris, J. J. Sutachan, G. J. V. Montoya, T. J. J. Blanck, E. Recio-Pinto, *Front. Mol. Neurosci.* **2013**, *6*, 23.

[S5]M. Feuerstein, E. Chleilat, S. Khakipoor, K. Michailidis, C. Ophoven, E. Roussa, *Cell Tissue Res.* **2017**, *370*, 211.

[S6]P. Rodrigues, I. Macaya, S. Bazzocco, R. Mazzolini, E. Andretta, H. Dopeso, S. Mateo-Lozano, J. Bilic, F. Carton-Garcia, R. Nieto, L. Suarez-Lopez, E. Afonso, S. Landolfi, J. Hernandez-Losa, K. Kobayashi, S. Ramon y Cajal, J. Tabernero, N. C. Tebbutt, J. M. Mariadason, S. Schwartz, D. Arango, *Nat. Commun.* **2014**, *5*, 5458.

[S7]J. Laboureau, L. Dubertret, C. Lebreton-De Coster, B. Coulomb, *Exp. Dermatol.* **2004**, *13*, 70.

[S8]A. von Thun, C. Preisinger, O. Rath, J. P. Schwarz, C. Ward, N. Monsefi, J. Rodriguez, A. Garcia-Munoz, M. Birtwistle, W. Bienvenut, K. I. Anderson, W. Kolch, A. von Kriegsheim, *Mol. Cell Biol.* **2013**, *33*, 4526.

[S9]K. I. Katayama, J. Melendez, J. M. Baumann, J. R. Leslie, B. K. Chauhan, N. Nemkul, R. A. Lang, C. Y. Kuan, Y. Zheng, Y. Yoshida, *Proc. Natl. Acad. Sci. U. S. A.* **2011**, *108*, 7607.

[S10] A. Po, M. Silvano, E. Miele, C. Capalbo, A. Eramo, V. Salvati, M. Todaro, Z. M. Besharat, G. Catanzaro, D. Cucchi, S. Coni, L. Di Marcotullio, G. Canettieri, A. Vacca, G. Stassi, E. De Smaele, M. Tartaglia, I. Screpanti, R. De Maria, E. Ferretti, *Oncogene* **2017**, *36*, 4641.

[S11] J. Zhang, J. R. Shemezis, E. R. McQuinn, J. Wang, M. Sverdlov, A. Chenn, *Neural Dev.* **2013**, 8, 7.

[S12] N. Nusser, E. Gosmanova, Y. Zheng, G. Tigyi, *J. Biol. Chem.* **2002**, *277*, 35840.

[S13] R. D. York, H. Yao, T. Dillon, C. L. Ellig, S. P. Eckert, E. W. McCleskey, P. J. S. Stork, *Nature* **1998**, *392*, 622.

[S14] S. Kelly, T. M. Bliss, A. K. Shah, G. H. Sun, M. Ma, W. C. Foo, J. Masel, M. A. Yenari, I. L. Weissman, N. Uchida, T. Palmer, G. K. Steinberg, *Proc. Natl. Acad. Sci. U. S. A.* **2004**, *101*, 11839.
